# Supplementary material for: Evaluation of tuberculosis diagnostic test accuracy using Bayesian latent class analysis in the presence of conditional dependence between the diagnostic tests used in a community-based tuberculosis screening study
Source: PLoS One. 2023 Mar 2;18(3):e0282417. doi: 10.1371/journal.pone.0282417 (PMC9980779; doi:10.1371/journal.pone.0282417)
Supplement: S1 File — (PDF) [file pone.0282417.s002.pdf]

Evaluation of TB diagnostic test accuracy using Bayesian latent class analysis in the presence of conditional dependence between the diagnostic tests used in a community-based TB screening study

Supplementary material

1. Model

Let the random variable $Y_j, j = 1,2, \dots, J$ denote the jth diagnostic test and the random variable D denote the latent disease status such that $Y_j = 0(1)$ if the jth diagnostic test result is negative (positive) and $D = 0(1)$ if the true disease status is negative (positive). Under the assumption of conditional independence, the joint probability of a combination of test results from a set of J diagnostic tests $\boldsymbol{Y} = (Y_1, Y_2, \cdots, Y_J)$ is given by [1]

$$Pr(\boldsymbol{Y}) = \sum_{d=0}^{1} Pr(D = d) \prod_{j=1}^{J} Pr(Y_j = y_j | D = d) \dots\dots\dots\dots\dots\dots\dots\dots\dots\dots\dots\dots\dots \quad (1)$$

However, for J dependent diagnostic tests, $Pr(\boldsymbol{Y})$ can be expressed using the chain rule of conditional probability as follows

$$Pr(\boldsymbol{Y}) = \sum_{d=0}^{1} Pr(D = d) \prod_{j=1}^{J} Pr\left(Y_j = y_j |\, D = d \bigcap_{j'=1|j>1}^{j-1} Y_{j'=y_{j'}} \right)$$

$$= \sum_{d=0}^{1} \theta_d \prod_{j=1}^{J} {p_{dj}}^{y_j} (1 - p_{dj})^{1-y_j} \dots\dots\dots\dots\dots\dots\dots\dots\dots\dots\dots.\dots\dots\dots \quad (2)$$

where

$$p_{dj} = Pr\left(Y_j = 1 |\, D = d \bigcap_{j'=1|j>1}^{j-1} Y_{j'=y_{j'}} \right) \text{and } \theta_d = Pr(D = d), d \in \{0,1\}$$

The probabilities p_{dj} and $\theta_d = Pr(D = d), d \in \{0,1\}$ can be calculated using regression methods.

Consequently,

$$Pr(Y_j = 1 | D = d) = \sum_{Y_{j-1} \in (0,1)} \cdots \sum_{Y_2 \in (0,1)} \sum_{Y_1 \in (0,1)} \prod_{k=1}^{j} p_{dk} \dots\dots\dots\dots\dots\dots\dots\dots \quad (3)$$

and $Pr(Y_j = 0|D = d) = 1 - Pr(Y_j = 1|D = d)$.

The model can be extended to include covariates known to affect the diagnostic accuracy and/or prevalence such that for a binary variable X, $Pr(\boldsymbol{Y})$ can be re-expressed as

$$Pr(\boldsymbol{Y}) = \sum_{d=0}^{1}\sum_{x=0}^{1} Pr(X = x)Pr(D = d|X = x)\prod_{j=1}^{J} Pr\left(Y_j = y_j|\ X = x \bigcap D = d \bigcap_{j'=1|j>1}^{j-1} Y_{j'=y_{j'}}\right)$$

For a vector $\boldsymbol{X}$ of p covariates, $Pr(\boldsymbol{X}) = Pr(X_1, X_2, \dots, X_p) = Pr(X_1)Pr(X_2|X_1) \dots Pr(X_p|X_1, X_2, \dots X_{p-1})$.

1.1. Bayesian Inference

Since the likelihood function of $\boldsymbol{y_i} = (y_{i1}, y_{i2}, \cdots, y_{iJ})$ depends on the (latent) disease status $d_i, i = 1,2,3, \cdots, N$, we have

$$Pr(\boldsymbol{y_i}, d_i|\theta, p_{d_ij}) = \left(\theta\prod_{j=1}^{J} p_{d_ij}^{y_{ij}}(1 - p_{d_ij})^{1-y_{ij}}\right)^{d_i}\left((1-\theta)\prod_{j=1}^{J} p_{d_ij}^{y_{ij}}(1 - p_{d_ij})^{1-y_{ij}}\right)^{1-d_i} \quad \dots\dots\dots (4)$$

We will only focus on the case where we have data on J diagnostic tests. Extension to incorporate covariates is straight forward. From (4) we have J conditional probability models to fit to determine $p_{d_ij}, j = 1,2, \dots J$. Since the outcome is Bernoulli distributed and p_{d_ij} is related to a set of (binary) independent variables we can define a binary regression model as $p_{id_ij} = H(\boldsymbol{y_i^T\beta_{dj}})$, where $\boldsymbol{y_i^T} = (y_{i0}, y_{i1}, \cdots, y_{ij})$ is a $(j+1) \times 1$ vector of observed variables (or diagnostic tests) with $y_{i0} = 1$, $\boldsymbol{\beta_{dj}}$ is a $(j+1) \times 1$ vector of unknown parameters to be estimated, and $H(.)$ is a known cumulative distribution function (CDF) linking the probabilities p_{id_ij} with the linear component $\boldsymbol{y_i^T\beta_{dj}}$ [2]. The unknown parameters $\boldsymbol{\beta_{dj}}$ were assigned $g(\boldsymbol{\beta_{dj}}) = N(\boldsymbol{\mu_{dj}}, \boldsymbol{\sigma_{dj}^2}I_{j+1})$ priors, where I_{j+1} is an identity matrix of dimension $(j+1)$ and $\boldsymbol{\sigma_{dj}^2}$ is a $(j+1) \times 1$ vector of variances among subjects whose true PTB status is d. The variances may not necessarily be the same (Tables S1 – S5). Similarly, the unobserved PTB status is a Bernoulli distributed latent variable. Therefore, the probability θ_i that the ith subject has PTB is related to a constant or a (set of) covariate(s) as follows: $\theta_i = H(\boldsymbol{x_i^T\omega})$, where $\boldsymbol{x_i^T} = (x_{i0}, x_{i2}, \cdots, x_{ip})$ is a $(p+1) \times 1$ vector of observed covariates $(x_{i0} = 1)$, $\boldsymbol{\omega}$ is a $(p+1) \times 1$ vector of unknown parameters to be estimated, and $H(.)$ is a known CDF

linking the probabilities θ_i with the linear component $\boldsymbol{x_i^T \omega}$. The unknown parameter(s) $\boldsymbol{\omega}$ were assigned $g(\boldsymbol{\omega}) = N(\boldsymbol{\mu_\theta}, \boldsymbol{\sigma_\theta^2 I}_{p+1})$ priors, where I_{p+1} is an identity matrix of dimension $p+1$ and $\boldsymbol{\sigma_\theta^2}$ is a $(p+1) \times 1$ vector of variances (for now we are working with $p+1=1$). Thus, the posterior distribution $g(\boldsymbol{\omega}, \boldsymbol{\beta_{dj}} \mid \boldsymbol{y_i}, d)$ is proportional to

$$g(\boldsymbol{\omega}) \prod_{i=1}^{N} \left(H(\boldsymbol{x_i^T \omega})^{d_i} \left(1 - H(\boldsymbol{x_i^T \omega})\right)^{1-d_i} \right) \times \left(\prod_{j=1}^{J} g(\boldsymbol{\beta_{1j}}) \prod_{i=1}^{N} \left(H(\boldsymbol{y_i^T \beta_{1j}}) \right)^{y_{ij} d_i} \left(1 - H(\boldsymbol{y_i^T \beta_{1j}})\right)^{(1-y_{ij}) d_i} \right)$$

$$\times \left(\prod_{j=1}^{J} g(\boldsymbol{\beta_{0j}}) \prod_{i=1}^{N} \left(H(\boldsymbol{y_i^T \beta_{0j}}) \right)^{y_{ij(1-d_i)}} \left(1 - H(\boldsymbol{y_i^T \beta_{0j}})\right)^{(1-y_{ij})(1-d_i)} \right) \dots \dots \dots \dots (5)$$

where $\int g(\boldsymbol{\beta_{dj}}) \prod_{i=1}^{N} \left(H(\boldsymbol{y_i^T \beta_{dj}})\right)^{y_{ij}} \left(1 - H(\boldsymbol{y_i^T \beta_{dj}})\right)^{1-y_{ij}} d\boldsymbol{\beta_{dj}}$, $d\epsilon\{0,1\}$ and $\int g(\boldsymbol{\omega}) \prod_{i=1}^{N} \left(H(\boldsymbol{x_i^T \omega})\right)^{y_{ij}} \left(1 - H(\boldsymbol{x_i^T \omega})\right)^{1-y_{ij}} d\boldsymbol{\omega}$ are not easy to determine analytically. Thus, the unknown parameters $\boldsymbol{\omega}$, and $\boldsymbol{\beta_{dj}}$, $j = 1,2,3, \dots, J$ will be estimated using Markov Chain Monte Carlo (MCMC) approach.

2. Prior distributions

Table S1 presents the models and the prior distributions for the parameters in the probit regression models for the analysis of CPTB study. Similarly, Tables S4 – S5 present the models and the prior distributions for the parameters in the probit regression models for the analysis of Vukuzazi study. The assumptions made on the potential dependencies for all the models were presented in the analysis section of the main document.

2.1. CPTB Data

In the analysis of CPTB data, we allowed TST (Y_1), radiography (Y_2), microscopy (Y_3), Xpert (Y_4) and culture (Y_5) as the possible ordering of the diagnostic tests.

Table S1: Prior distributions for the parameters in the probit regression models for childhood pulmonary TB data

Response	Model structure	Model 0	Model 1	Model 2	Model 3	Model 4
D	$Pr(D = 1) = \emptyset(\omega)$	$g(\omega)$~N(0,1)	$g(\omega)$~N(0,1)	$g(\omega)$~N(0,1)	$g(\omega)$~N(0,1)	$g(\omega)$~N(0,1)
Y_1	$Pr(Y_{i1} = 1 \vert D_i = 1) = \emptyset(\beta_{11})$	$g(\beta_{11})$~N(0,10)	$g(\beta_{11})$~N(0,10)	$g(\beta_{11})$~N(0,10)	$g(\beta_{11})$~N(0,10)	$g(\beta_{11})$~N(0,10)
	$Pr(Y_{i1} = 1 \vert D_i = 0) = \emptyset(\alpha_{11})$	$g(\alpha_{11})$~N(0,10)	$g(\alpha_{11})$~N(0,10)	$g(\alpha_{11})$~N(0,10)	$g(\alpha_{11})$~N(0,10)	$g(\alpha_{11})$~N(0,10)
Y_2	$Pr\ (Y_{i2} = 1 \vert Y_{i1}, D_i{=}1) = \emptyset(\beta_{12} + \beta_{22} y_{i1})$	$g(\beta_{12})$~N(0,10)<br>-	$g(\beta_{12})$~N(0,10)<br>-	$g(\beta_{12})$~N(0,10)<br>-	$g(\beta_{12})$~N(0,10)<br>$g(\beta_{22})$~N(0,10)	$g(\beta_{12})$~N(0,10)<br>$g(\beta_{22})$~N(0,10)
	$Pr\ (Y_{i2} = 1 \vert Y_{i1}, D_i{=}0) = \emptyset(\alpha_{12} + \alpha_{22} y_{i1})$	$g(\alpha_{12})$~N(0,10)<br>-	$g(\alpha_{12})$~N(0,10)<br>-	$g(\alpha_{12})$~N(0,10)<br>$g(\alpha_{22})$~N(0,10)	$g(\alpha_{12})$~N(0,10)<br>$g(\alpha_{22})$~N(0,10)	$g(\alpha_{12})$~N(0,10)<br>-
Y_3	$Pr(Y_{i3} = 1 \vert Y_{i1}, Y_{i2}, D_i{=}1)$<br>$= \emptyset(\beta_{13} + \beta_{23} y_{i1} + \beta_{33} y_{i2})$	$g(\beta_{13})$~N(0,10)<br>-<br>-	$g(\beta_{13})$~N(0,10)<br>$g(\beta_{23})$~N(0,10)<br>-	$g(\beta_{13})$~N(0,10)<br>$g(\beta_{23})$~N(0,10)<br>-	$g(\beta_{13})$~N(0,10)<br>$g(\beta_{23})$~N(0,10)<br>$g(\beta_{33})$~N(0,10)	$g(\beta_{13})$~N(0,10)<br>$g(\beta_{23})$~N(0,10)<br>$g(\beta_{33})$~N(0,10)
	$Pr\ (Y_{i3} = 1 \vert D_i{=}0) = \emptyset(\alpha_{13})$	$g(\alpha_{13})$~N(-3, 0.1)	$g(\alpha_{13})$~N(-3,0.1)	$g(\alpha_{13})$~N(-3,0.1)	$g(\alpha_{13})$~N(-3,0.1)	$g(\alpha_{13})$~N(-3,0.1)
Y_4	$Pr(Y_{i4} = 1 \vert Y_{i1}, Y_{i2}, Y_{i3}, D_i{=}1)$<br>$= \emptyset(\beta_{14} + \beta_{24} y_{i1} + \beta_{34} y_{i2} + \beta_{44} y_{i3})$	$g(\beta_{14})$~N(0,10)<br>-<br>-<br>-	$g(\beta_{14})$~N(0,10)<br>$g(\beta_{24})$~N(0,10)<br>-<br>$g(\beta_{44})$~N(0,10)	$g(\beta_{14})$~N(0,10)<br>$g(\beta_{24})$~N(0,10)<br>-<br>$g(\beta_{44})$~N(0,10)	$g(\beta_{14})$~N(0,10)<br>$g(\beta_{24})$~N(0,10)<br>$g(\beta_{34})$~N(0,10)<br>$g(\beta_{44})$~N(0,10)	$g(\beta_{14})$~N(0,10)<br>$g(\beta_{24})$~N(0,10)<br>$g(\beta_{34})$~N(0,10)<br>$g(\beta_{44})$~N(0,10)
	$Pr\ (Y_{i4} = 1 \vert D_i{=}0) = \emptyset(\alpha_{14})$	$g(\alpha_{14})$~N(-3,0.1)	$g(\alpha_{14})$~N(-3,0.1)	$g(\alpha_{14})$~N(-3,0.1)	$g(\alpha_{14})$~N(-3,0.1)	$g(\alpha_{14})$~N(-3,0.1)
Y_5	$Pr(Y_{i5} = 1 \vert Y_{i1}, Y_{i2}, Y_{i3}, Y_{i4}, D_i{=}1)$<br>$= \emptyset(\beta_{15} + \beta_{25} y_{i1} + \beta_{35} y_{i2} + \beta_{45} y_{i3} + \beta_{55} y_{i4})$	$g(\beta_{15})$~N(0,10)<br>-<br>-<br>-<br>-	$g(\beta_{15})$~N(0,10)<br>$g(\beta_{25})$~N(0,10)<br>-<br>$g(\beta_{45})$~N(0,10)<br>$g(\beta_{55})$~N(0,10)	$g(\beta_{15})$~N(0,10)<br>$g(\beta_{25})$~N(0,10)<br>-<br>$g(\beta_{45})$~N(0,10)<br>$g(\beta_{55})$~N(0,10)	$g(\beta_{15})$~N(0,10)<br>$g(\beta_{25})$~N(0,10)<br>$g(\beta_{35})$~N(0,10)<br>$g(\beta_{45})$~N(0,10)<br>$g(\beta_{55})$~N(0,10)	$g(\beta_{15})$~N(0,10)<br>$g(\beta_{25})$~N(0,10)<br>$g(\beta_{35})$~N(0,10)<br>$g(\beta_{45})$~N(0,10)<br>$g(\beta_{55})$~N(0,10)
	$Pr(Y_{i5} = 1 \vert D_i{=}0) = \emptyset(\alpha_{15})$	$g(\alpha_{15})$~N(-3,0.1)	$g(\alpha_{15})$~N(-3,0.1)	$g(\alpha_{15})$~N(-3,0.1)	$g(\alpha_{15})$~N(-3,0.1)	$g(\alpha_{15})$~N(-3,0.1)

D – Disease (CPTB), Y_1 – TST, Y_2 – Radiography, Y_3 – Smear microscopy, Y_4 – Xpert MTB/RIF, Y_5 – Culture, "-" implies the parameter is not included (i.e set to zero) in the model, Model 0 was based on the assumption of conditional independence, Model 1 was based on expert opinion [3], Model 2 adds dependency between TST and radiography in non-CPTB cases to Model 1, Model 3 additionally allows dependency of all diagnostic tests with radiography among the true CPTB cases. Model 4 is the reduced version of Model 3 that omits the dependence between TST and radiography in non-CPTB cases

Table S2: Prior distributions for the parameters in the probit regression models for Vukuzazi data

	Model structure	Model 0	Model 1	Model 2
D	$Pr(D = 1) = \emptyset(\omega)$	$g(\omega)$~N(-3,0.1)	$g(\omega)$~N(-3,0.1)	$g(\omega)$~N(-3,0.1)
Y_1	$Pr(Y_{i1} = 1 \| D_i = 1)$ $= \emptyset(\beta_{11})$	$g(\beta_{11})$~N(0,0.1)	$g(\beta_{11})$~N(0,0.1)	$g(\beta_{11})$~N(0,0.1)
	$Pr(Y_{i1} = 1 \| D_i = 0)$ $= \emptyset(\alpha_{11})$	$g(\alpha_{11})$~N(0,10)	$g(\alpha_{11})$~N(0,10)	$g(\alpha_{11})$~N(0,10)
Y_2	$Pr(Y_{i2} = 1 \| D_i{=}1) = \emptyset(\beta_{12})$	$g(\beta_{12})$~N(0,0.1)	$g(\beta_{12})$~N(0,0.1)	$g(\beta_{12})$~N(0,0.1)
	$Pr(Y_{i2} = 1 \| Y_{i1}, D_i{=}0) =$ $\emptyset(\alpha_{12} + \alpha_{22} y_{i1})$	$g(\alpha_{12})$~N(0,10)<br>-	$g(\alpha_{12})$~N(0,10)<br>-	$g(\alpha_{12})$~N(0,10)<br>$g(\alpha_{22})$~N(0,10)
Y_3	$Pr(Y_{i3} = 1 \| Y_{i2}, D_i{=}1)$ $= \emptyset(\beta_{13} + \beta_{23} y_{i2})$	$g(\beta_{13})$~N(0,0.1)<br>-	$g(\beta_{13})$~N(0,0.1)<br>$g(\beta_{23})$~N(0,0.1)	$g(\beta_{13})$~N(0,0.1)<br>$g(\beta_{23})$~N(0,0.1)
	$Pr(Y_{i3} = 1 \| Y_{i1}, Y_{i2}, D_i{=}0)$ $= \emptyset(\alpha_{13} + \alpha_{23} y_{i1} + \alpha_{33} y_{i2})$	$g(\alpha_{13})$~N(0,10)	$g(\alpha_{13})$~N(0,10)<br>-	$g(\alpha_{13})$~N(0,10)<br>$g(\alpha_{23})$~N(0,10)<br>$g(\alpha_{33})$~N(0,10)
Y_4	$Pr(Y_{i4} = 1 \| Y_{i2}, Y_{i3}, D_i{=}1)$ $= \emptyset(\beta_{14} + \beta_{24} y_{i2} + \beta_{34} y_{i3})$	$g(\beta_{14})$~N(0,0.1)<br>-	$g(\beta_{14})$~N(0,0.1)<br>$g(\beta_{24})$~N(0,0.1)<br>$g(\beta_{34})$~N(0,0.1)	$g(\beta_{14})$~N(0,0.1)<br>$g(\beta_{24})$~N(0,0.1)<br>$g(\beta_{34})$~N(0,0.1)
	$Pr(Y_{i4} = 1 \| Y_{i1}, Y_{i2}, Y_{i3}, D_i{=}0)$ $= \emptyset\begin{pmatrix}\alpha_{14} + \alpha_{24} y_{i1} + \\ \alpha_{34} y_{i2} + \alpha_{44} y_{i3}\end{pmatrix}$	$g(\alpha_{14})$~N(0,10)<br>-	$g(\alpha_{14})$~N(0,10)<br>-	$g(\alpha_{14})$~N(0,10)<br>$g(\alpha_{24})$~N(0,10)<br>$g(\alpha_{34})$~N(0,10)<br>$g(\alpha_{44})$~N(0,10)
Y_5	$Pr(Y_{i5} = 1 \| D_i{=}1) = \emptyset(\beta_{15})$	$g(\beta_{15})$~N(0,0.1)	$g(\beta_{15})$~N(0,0.1)	$g(\beta_{15})$~N(0,0.1)
	$Pr(Y_{i5} = 1 \| D_i{=}0)$ $= \emptyset(\alpha_{15})$	$g(\alpha_{15})$~N(-1.88,0.01)<br>-	$g(\alpha_{15})$~N(-1.88,0.01)<br>-	$g(\alpha_{15})$~N(-1.88,0.01)<br>-
Y_6	$Pr(Y_{i6} = 1 \| D_i{=}1)$ $= \emptyset(\beta_{16} + \beta_{26} y_{i5})$	$g(\beta_{16})$~N(0,0.1)<br>-	$g(\beta_{16})$~N(0.84,0.1)<br>$g(\beta_{26})$~N(0,0.1)	$g(\beta_{16})$~N(0.84,0.1)<br>$g(\beta_{26})$~N(0,0.1)
	$\mathrm{Pr}(Y_{i6} = 1 \| D_i{=}0)$ $= \emptyset(\alpha_{16})$	$g(\alpha_{16})$~N(-3.09,0.01)	$g(\alpha_{16})$~N(-3.09,0.01)	$g(\alpha_{16})$~N(-3.09,0.01)

D – Disease (PTB), Y_1 – any TB symptom, Y_2 – Radiologist conclusion, Y_3 – CAD4TBv5≥53 Y_4 – CAD4TBv6≥53, Y_5 – Xpert Ultra, Y_6 – Culture, "-" implies the parameter is not included (i.e set to zero) in the model, Model 0 – Based on the assumption of conditional independence, Model 1 – Accounts for conditional dependence between radiologist conclusion, CAD4TBv5≥53 and CAD4TBv6≥53 and between Xpert Ultra and culture among the PTB cases and allows conditional independence between all the diagnostic tests among non-PTB cases, Model 2 – Accounts for conditional dependence between radiologist conclusion, CAD4TBv5≥53 and CAD4TBv6≥53 and between Xpert Ultra and culture among the PTB cases and conditional dependence between any TB symptom, radiologist conclusion, CAD4TBv5≥53 and CAD4TBv6≥53 among non-PTB cases

Table S3: Prior distributions for the parameters in the probit regression models for Vukuzazi data

	Model structure	Model 3
D	$Pr(D=1)=\emptyset(\omega)$	$g(\omega)$~N(-3,0.1)
Y_1	$Pr(Y_{i1}=1\|D_i=1)=\emptyset(\beta_{11})$	$g(\beta_{11})$~N(0,0.1)
	$Pr(Y_{i1}=1\|D_i=0)=\emptyset(\alpha_{11})$	$g(\alpha_{11})$~N(0,10)
Y_2	$Pr(Y_{i2}=1\|Y_{i1},D_i{=}1)=\emptyset(\beta_{12})$	$g(\beta_{12})$~N(0,0.1)
	$Pr(Y_{i2}=1\|Y_{i1},D_i{=}0)=\emptyset(\alpha_{12}+\alpha_{22}y_{i1})$	$g(\alpha_{12})$~N(0,10)<br>$g(\alpha_{22})$~N(0,10)
Y_3	$Pr(Y_{i3}=1\|Y_{i2},D_i{=}1)=\emptyset(\beta_{13}+\beta_{23}y_{i2})$	$g(\beta_{13})$~N(0,0.1)<br>$g(\beta_{23})$~N(0,0.1)
	$\Pr(Y_{i3}=1\|Y_{i1},Y_{i2},D_i{=}0)=\emptyset(\alpha_{13}+\alpha_{23}y_{i1}+\alpha_{33}y_{i2})$	$g(\alpha_{13})$~N(0,10)<br>$g(\alpha_{23})$~N(0,10)<br>$g(\alpha_{33})$~N(0,10)
Y_4	$Pr(Y_{i4}=1\|Y_{i2},Y_{i3},D_i{=}1)=\emptyset(\beta_{14}+\beta_{24}y_{i2}+\beta_{34}y_{i3})$	$g(\beta_{14})$~N(0,0.1)<br>$g(\beta_{24})$~N(0,0.1)<br>$g(\beta_{34})$~N(0,0.1)
	$\Pr(Y_{i4}=1\|Y_{i1},Y_{i2},Y_{i3},D_i{=}0)=\emptyset(\alpha_{14}+\alpha_{24}y_{i1}+\alpha_{34}y_{i2}+\alpha_{44}y_{i3})$	$g(\alpha_{14})$~N(0,10)<br>$g(\alpha_{24})$~N(0,10)<br>$g(\alpha_{34})$~N(0,10)<br>$g(\alpha_{44})$~N(0,10)
Y_5	$Pr(Y_{i5}=1\|Y_{i2},Y_{i3},Y_{i4},D_i{=}1)=\emptyset(\beta_{15}+\beta_{25}y_{i2}+\beta_{35}y_{i3}+\beta_{45}y_{i4})$	$g(\beta_{15})$~N(0,0.1)<br>$g(\beta_{25})$~N(0,0.1)<br>$g(\beta_{35})$~N(0,0.1)<br>$g(\beta_{45})$~N(0,0.1)
	$Pr(Y_{i5}=1\|D_i{=}0)=\emptyset(\alpha_{15})$	$g(\alpha_{15})$~N(-1.88,0.01)
Y_6	$Pr(Y_{i6}=1\|Y_{i2},Y_{i3},Y_{i4},Y_{i5},D_i{=}1)=\emptyset(\beta_{16}+\beta_{26}y_{i2}+\beta_{36}y_{i3}+\beta_{46}y_{i4}+\beta_{56}y_{i5})$	$(\beta_{16})\sim N(0.84,0.1)$<br>$g(\beta_{26})\sim N(0,0.1)$<br>$g(\beta_{36})\sim N(0,0.1)$<br>$g(\beta_{46})\sim N(0,0.1)$<br>$g(\beta_{56})\sim N(0,0.1)$
	$Pr(Y_{i6}=1\|D_i{=}0)=\emptyset(\alpha_{16})$	$g(\alpha_{16})$~N(-3.09,0.01)

D – Disease (PTB), Y_1 – any TB symptom, Y_2 – Radiologist conclusion, Y_3 – CAD4TBv5≥53 Y_4 – CAD4TBv6≥53, Y_5 – Xpert Ultra, Y_6 – Culture, Model 3 – Accounts for conditional dependence between all the diagnostic tests except any TB symptom among the PTB cases and dependence between any TB symptom, radiologist conclusion, CAD4TBv5≥53 and CAD4TBv6≥53 among non-PTB cases.

Table S4: Prior distributions for the parameters in the probit regression models for Vukuzazi data

	Model structure	Model 4
D	$Pr(D = 1) = \emptyset(\omega)$	$g(\omega)$~N(-3,0.1)
Y_1	$Pr(Y_{i1} = 1 \mid D_i = 1) = \emptyset(\beta_{11})$	$g(\beta_{11})$~N(0,0.1)
	$Pr(Y_{i1} = 1 \mid D_i = 0) = \emptyset(\alpha_{11})$	$g(\alpha_{11})$~N(0,10)
Y_2	$Pr(Y_{i2} = 1 \mid Y_{i1}, D_i{=}1) = \emptyset(\beta_{12} + \beta_{22} y_{i1})$	$g(\beta_{12})$~N(0,0.1)<br>$g(\beta_{22})$~N(0,0.1)
	$Pr(Y_{i2} = 1 \mid Y_{i1}, D_i{=}0) = \emptyset(\alpha_{12} + \alpha_{22} y_{i1})$	$g(\alpha_{12})$~N(0,10)<br>$g(\alpha_{22})$~N(0,10)
Y_3	$Pr(Y_{i3} = 1 \mid Y_{i1}, Y_{i2}, D_i{=}1) = \emptyset(\beta_{13} + \beta_{23} y_{i1} + \beta_{33} y_{i2})$	$g(\beta_{13})$~N(0,0.1)<br>$g(\beta_{23})$~N(0,0.1)<br>$g(\beta_{33})$~N(0,0.1)
	$\Pr(Y_{i3} = 1 \mid Y_{i1}, Y_{i2}, D_i{=}0) = \emptyset(\alpha_{13} + \alpha_{23} y_{i1} + \alpha_{33} y_{i2})$	$g(\alpha_{13})$~N(0,10)<br>$g(\alpha_{23})$~N(0,10)<br>$g(\alpha_{33})$~N(0,10)
Y_4	$Pr(Y_{i4} = 1 \mid Y_{i1}, Y_{i2}, Y_{i3}, D_i{=}1) = \emptyset(\beta_{14} + \beta_{24} y_{i1} + \beta_{34} y_{i2} + \beta_{44} y_{i3})$	$g(\beta_{14})$~N(0,0.1)<br>$g(\beta_{24})$~N(0,0.1)<br>$g(\beta_{34})$~N(0,0.1)<br>$g(\beta_{44})$~N(0,0.1)
	$\Pr(Y_{i4} = 1 \mid Y_{i1}, Y_{i2}, Y_{i3}, D_i{=}0) = \emptyset(\alpha_{14} + \alpha_{24} y_{i1} + \alpha_{34} y_{i2} + \alpha_{44} y_{i3})$	$g(\alpha_{14})$~N(0,10)<br>$g(\alpha_{24})$~N(0,10)<br>$g(\alpha_{34})$~N(0,10)<br>$g(\alpha_{44})$~N(0,10)
Y_5	$Pr(Y_{i5} = 1 \mid Y_{i1}, Y_{i2}, Y_{i3}, Y_{i4}, D_i{=}1) = \emptyset(\beta_{15} + \beta_{25} y_{i1} + \beta_{35} y_{i2} + \beta_{45} y_{i3} + \beta_{55} y_{i4})$	$g(\beta_{15})$~N(0,0.1)<br>$g(\beta_{25})$~N(0,0.1)<br>$g(\beta_{35})$~N(0,0.1)<br>$g(\beta_{45})$~N(0,0.1)<br>$g(\beta_{55})$~N(0,0.1)
	$Pr(Y_{i5} = 1 \mid D_i{=}0) = \emptyset(\alpha_{15})$	$g(\alpha_{15})$~N(-1.88,0.01)
Y_6	$Pr(Y_{i6} = 1 \mid Y_{i1}, Y_{i2}, Y_{i3}, Y_{i4}, Y_{i5}, D_i{=}1)$<br>$= \emptyset(\beta_{16} + \beta_{26} y_{i1} + \beta_{36} y_{i2} + \beta_{46} y_{i3} + \beta_{56} y_{i4} + \beta_{66} y_{i5})$	$g(\beta_{16})$~N(0.84,0.1)<br>$g(\beta_{26})$~N(0, 0.1)<br>$g(\beta_{36})$~N(0, 0.1)<br>$g(\beta_{46})$~N(0, 0.1)<br>$g(\beta_{56})$~N(0, 0.1)<br>$g(\beta_{66})$~N(0,0.1)
	$Pr(Y_{i6} = 1 \mid D_i{=}0) = \emptyset(\alpha_{16})$	$g(\alpha_{16})$~N(-3.09,0.01)

D – Disease (PTB), Y_1 – any TB symptom, Y_2 – Radiologist conclusion, Y_3 – CAD4TBv5≥53 Y_4 – CAD4TBv6≥53, Y_5 – Xpert Ultra, Y_6 – Culture, Model 4 – Accounts for conditional dependence between all the diagnostic tests among the PTB cases and conditional dependence between any TB symptom, radiologist conclusion, CAD4TBv5≥53 and CAD4TBv6≥53 among non-PTB cases

Table S5: Prior distributions for the parameters in the probit regression models for Vukuzazi data

	Model structure	Model 2 with covariates
D	$Pr(D=1\|X_{i1},X_{i2},X_{i3}) = \emptyset\begin{pmatrix}\omega_0+\omega_1 x_{i3}+\omega_2 x_{i2}+\omega_3 I(x_{i1}=2)+\\ \omega_4 I(x_{i1}=3)+\omega_5 I(x_{i1}=4)\end{pmatrix}$	$g(\omega_0)$~N(-5,0.1) $g(\omega_1)$~N(0,0.01), …, $g(\omega_5)$~N(0,0.01)
Y_1	$Pr(Y_{i1}=1\|X_{i1},X_{i2},X_{i3},D_i=1) = \emptyset\begin{pmatrix}\beta_{11}+\beta_{21}x_{i3}+\beta_{31}x_{i2}+\beta_{41}I(x_{i1}=2)+\\ \beta_{51}I(x_{i1}=3)+\beta_{61}I(x_{i1}=4)\end{pmatrix}$	$g(\beta_{11})$~N(0,10) $g(\beta_{21})\ldots g(\beta_{61})$~N(0,1)
	$Pr(Y_{i1}=1\|X_{i1},X_{i2},X_{i3},D_i=0) = \emptyset\begin{pmatrix}\alpha_{11}+\alpha_{21}x_{i3}+\alpha_{31}x_{i2}+\alpha_{41}I(x_{i1}=2)\\ +\alpha_{51}I(x_{i1}=3)+\alpha_{61}I(x_{i1}=4)\end{pmatrix}$	$g(\alpha_{11})$~N(0,10) $g(\alpha_{21})\ldots g(\alpha_{61})$~N(0,1)
Y_2	$Pr(Y_{i2}=1\|X_{i1},X_{i2},X_{i3},D_i=1) = \emptyset\begin{pmatrix}\beta_{12}+\beta_{22}x_{i3}+\beta_{32}x_{i2}+\beta_{42}I(x_{i1}=2)+\\ \beta_{52}I(x_{i1}=3)+\beta_{62}I(x_{i1}=4)\end{pmatrix}$	$g(\beta_{12})\ldots g(\beta_{62})$~N(0,1)
	$Pr(Y_{i2}=1\|Y_{i1},X_{i1},X_{i2},X_{i3},D_i=0) = \emptyset\begin{pmatrix}\alpha_{12}+\alpha_{22}y_{i1}+\alpha_{32}x_{i3}+\alpha_{42}x_{i2}+\alpha_{52}I(x_{i1}=2)\\ +\alpha_{62}I(x_{i1}=3)+\alpha_{72}I(x_{i1}=4)\end{pmatrix}$	$g(\alpha_{12}), g(\alpha_{22})$~N(0,10) $g(\alpha_{32})\ldots g(\alpha_{72})$~N(0,1)
Y_3	$Pr(Y_{i3}=1\|Y_{i2},X_{i1},X_{i2},X_{i3},D_i=1) = \emptyset\begin{pmatrix}\beta_{13}+\beta_{23}y_{i2}+\beta_{33}x_{i3}+\beta_{43}x_{i2}+\beta_{53}I(x_{i1}=2)\\ +\beta_{63}I(x_{i1}=3)+\beta_{73}I(x_{i1}=4)\end{pmatrix}$	$g(\beta_{13})\ldots g(\beta_{73})$~N(0,1)
	$\Pr(Y_{i3}=1\|Y_{i1},Y_{i2},X_{i1},X_{i2},X_{i3},D_i=0) = \emptyset\begin{pmatrix}\alpha_{13}+\alpha_{23}y_{i1}+\alpha_{33}y_{i2}+\alpha_{43}x_{i3}+\alpha_{53}x_{i2}\\ +\alpha_{63}I(x_{i1}=2)+\alpha_{73}I(x_{i1}=3)+\alpha_{83}I(x_{i1}=4)\end{pmatrix}$	$g(\alpha_{13})\ldots g(\alpha_{33})$~N(0,10) $g(\alpha_{43})\ldots g(\alpha_{83})$~N(0,1)
Y_4	$Pr(Y_{i4}=1\|Y_{i2},Y_{i3},X_{i1},X_{i2},X_{i3},D_i=1) = \emptyset\begin{pmatrix}\beta_{14}+\beta_{24}y_{i2}+\beta_{34}y_{i3}+\beta_{44}x_{i3}+\beta_{54}x_{i2}\\ +\beta_{64}I(x_{i1}=2)+\beta_{74}I(x_{i1}=3)+\beta_{84}I(x_{i1}=4)\end{pmatrix}$	$g(\beta_{14})\ldots g(\beta_{84})$~N(0,1)
	$\Pr(Y_{i4}=1\|Y_{i1},Y_{i2},Y_{i3},X_{i1},X_{i2},X_{i3},D_i=0) = \emptyset\begin{pmatrix}\alpha_{14}+\alpha_{24}y_{i1}+\alpha_{34}y_{i2}+\alpha_{44}y_{i3}+\alpha_{54}x_{i3}+\alpha_{64}x_{i2}\\ +\alpha_{74}I(x_{i1}=2)+\alpha_{84}I(x_{i1}=3)+\alpha_{94}I(x_{i1}=4)\end{pmatrix}$	$g(\alpha_{14})\ldots g(\alpha_{44})$~N(0,10) $g(\alpha_{54})\ldots g(\alpha_{94})$~N(0,1)
Y_5	$Pr(Y_{i5}=1\|X_{i1},X_{i2},X_{i3},D_i=1) = \emptyset\begin{pmatrix}\beta_{15}+\beta_{25}x_{i3}+\beta_{35}x_{i2}+\beta_{45}I(x_{i1}=2)+\beta_{55}\\ I(x_{i1}=3)+\beta_{65}I(x_{i1}=4)\end{pmatrix}$	$g(\beta_{15})\ldots g(\beta_{65})$~N(0,1)
	$Pr(Y_{i5}=1\|D_i=0) = \emptyset(\alpha_{15})$	$g(\alpha_{15})$~N(-1.88,0.01)
Y_6	$Pr(Y_{i6}=1\|Y_{i5},X_{i1},X_{i2},X_{i3},D_i=1) = \emptyset\begin{pmatrix}\beta_{16}+\beta_{26}y_{i5}+\beta_{36}x_{i3}+\beta_{46}x_{i2}+\beta_{56}I(x_{i1}=2)+\\ \beta_{66}I(x_{i1}=3)+\beta_{76}I(x_{i1}=4)\end{pmatrix}$	$g(\beta_{16})$~N(0.84,0.1) $g(\beta_{26})\ldots g(\beta_{76})$~N(0,1)
	$Pr(Y_{i6}=1\|D_i=0) = \emptyset(\alpha_{16})$	$g(\alpha_{16})$~N(-3.09,0.01)

D – Disease (PTB), Y_1 – any TB symptom, Y_2 – Radiologist conclusion, Y_3 – CAD4TBv5 ≥53 Y_4 – CAD4TBv6 ≥53, Y_5 – Xpert Ultra, Y_6 – Culture; Model 2 – Accounts for conditional dependence between radiologist conclusion, CAD4TBv5≥53 and CAD4TBv6≥53 and between Xpert Ultra and culture among the PTB cases and conditional dependence between any TB symptom, radiologist conclusion, CAD4TBv5≥53 and CAD4TBv6≥53 among non-PTB cases, adjusted for covariates (age, sex and HIV status).

In Table S5, D denotes disease (PTB), Y_1, Y_2, Y_3, Y_4, Y_5 and Y_6 denotes any TB symptom , Radiologist conclusion, CAD4TBv5 ≥53, CAD4TBv6 ≥53, Xpert Ultra and Culture respectively. X_1 denotes age (categorical variable), X_2 denotes sex (Male/Female) and X_3 denotes HIV status (+/-)

The choice of the informative prior for the parameters of the probit regression models was adapted based on the expert opinion and previously published material. This was chosen in order to yield a posterior distribution that spans the range of plausible values for the parameter.

The joint probability of random variables Y and X_1, X_2, X_3 given by $Pr(Y, X_1, X_2, X_3)$ can be expressed using chain rule of conditional probabilities as $Pr(X_1)Pr(X_2|X_1)Pr(X_3|X_1, X_2)Pr(Y|X_1, X_2, X_3)$. Where Y is either the disease status or diagnostic test and X_1 – Age (four level categorical variable: 15-19 years, 30-49 years, 50-69 years, ≥70 years) with reference category as 15-19 years, X_2 - Sex (Male/Female), X_3 – HIV status (+/-). The conditional probabilities were computed using probit regression models with priors from Gaussian distribution. The unknown parameters ofX_1, X_2 and X_3 in the model $Pr(Y|X_1, X_2, X_3)$ are assigned priors from N(0,10) (Table S5). The unknown parameters of X_1 and X_2 in the models $Pr(X_2|X_1)$ and $Pr(X_3|X_1, X_2)$ are assigned priors from N(0,10). To model $Pr(X_1)$ we assigned non-informative dirichlet prior, $Dir(\vartheta_1 = 1, \vartheta_2 = 1, \vartheta_3 = 1, \vartheta_4 = 1)$, to the parameters of the multinomial distribution.

3. Computer-Aided Detection for TB (CAD4TB) Threshold

Given all the diagnostic tests in use are imperfect, the true TB status is unknown. This is the complexity in diagnostic studies without a perfect reference standard. However, as alluded to in the background of the abstract and in the introduction, this problem can be handled using latent class analysis, a statistical methodology that has been in use over the past four decades in many disciplines but scarcely used in the field of infectious diseases, including the field of TB. This method identifies unobserved mutually exclusive subgroups in the population using information from the measured subject characteristics. In our case, using the information (test results) from all the imperfect diagnostic tests the model can stochastically tease out the TB and non-TB cases. Consequently, using the derived subgroups, the sensitivity and specificity of the tests can be calculated.

CAD4TB thresholds were determined based on secondary analysis of data from the community-based multi-morbidity survey in KwaZulu-Natal, South Africa (“Vukuzazi” study) using Bayesian latent class analysis. In this survey, the digital chest X-ray images were interpreted using CAD4TB version 5 (CAD4TBv5) and CAD4TBv6. The thresholds for both CAD4TBv5 and CAD4TBv6 were determined by performing LCA to estimate the sensitivity and specificity of CAD4TB at integer thresholds S_k, k = 2, 3, …, K-1, for K distinct CAD4TB scores. Thus, there were K-2 models fit and for each model, the estimates of pulmonary TB prevalence and diagnostic test sensitivity and specificity were obtained. The estimates of sensitivity and specificity for CAD4TB were used to construct a receiver operating characteristic (ROC) curve to help identify a plausible CAD4TB cut-off score.

Determination of CAD4TB cut-off scores proceeded by first fitting a two-class latent class model with any TB symptom, radiologist interpretation (chest X-ray abnormality suggestive of active TB), CAD4TBv5≥S_k, Xpert Ultra (excluding trace) and culture. Using the estimates of sensitivity and specificity for CAD4TBv5 obtained from the fitted models we constructed a ROC curve and determined CAD4TBv5 threshold, say S_{t1}. Next, we fitted a two-class latent class model with any TB symptom, radiologist interpretation (chest X-ray abnormality suggestive of active TB), CAD4TBv5≥S_{t1}, CAD4TBv6≥S_k, Xpert Ultra (excluding trace) and culture, where we now set threshold for CAD4TBv5 at S_{t1}. Again, using the same approach, we determined the threshold for CAD4TBv6, say S_{t2}. For both CAD4TBv5 and CAD4TBv6, we aimed at a threshold score that produced sensitivity and specificity estimates close to 80%. The cut-off values were 53 for CAD4TBv5 and 52 for CAD4TBv6 (Fig S1). Nonetheless, we opted for the same cut-off of 53 for both CAD4TBv5 and CAD4TBv6 based on the lowess smoother that revealed overlap of the two curves at around 80% for both the sensitivity and specificity for CAD4TBv5 and CAD4TBv6. The cut-off score of 53 yielded a sensitivity of 83% and a specificity of 80% for CAD4TBv5. In the model that included CAD4TBv5≥53 and CAD4TBv6 the threshold score of 52 for CAD4TBv6 yielded a sensitivity of 84% and a specificity of 81% While a threshold score of 53 for CAD4TBv6 yielded a sensitivity and specificity of 80% and 83% respectively (Fig S1). This is no surprise given the distribution of the difference in scores between the two versions of CAD4TB that is centered around zero (Fig S2). Fig S1 shows the receiver operating characteristic (ROC) curves of the estimates of sensitivity and specificity for CAD4TBv5 and CAD4TBv6.

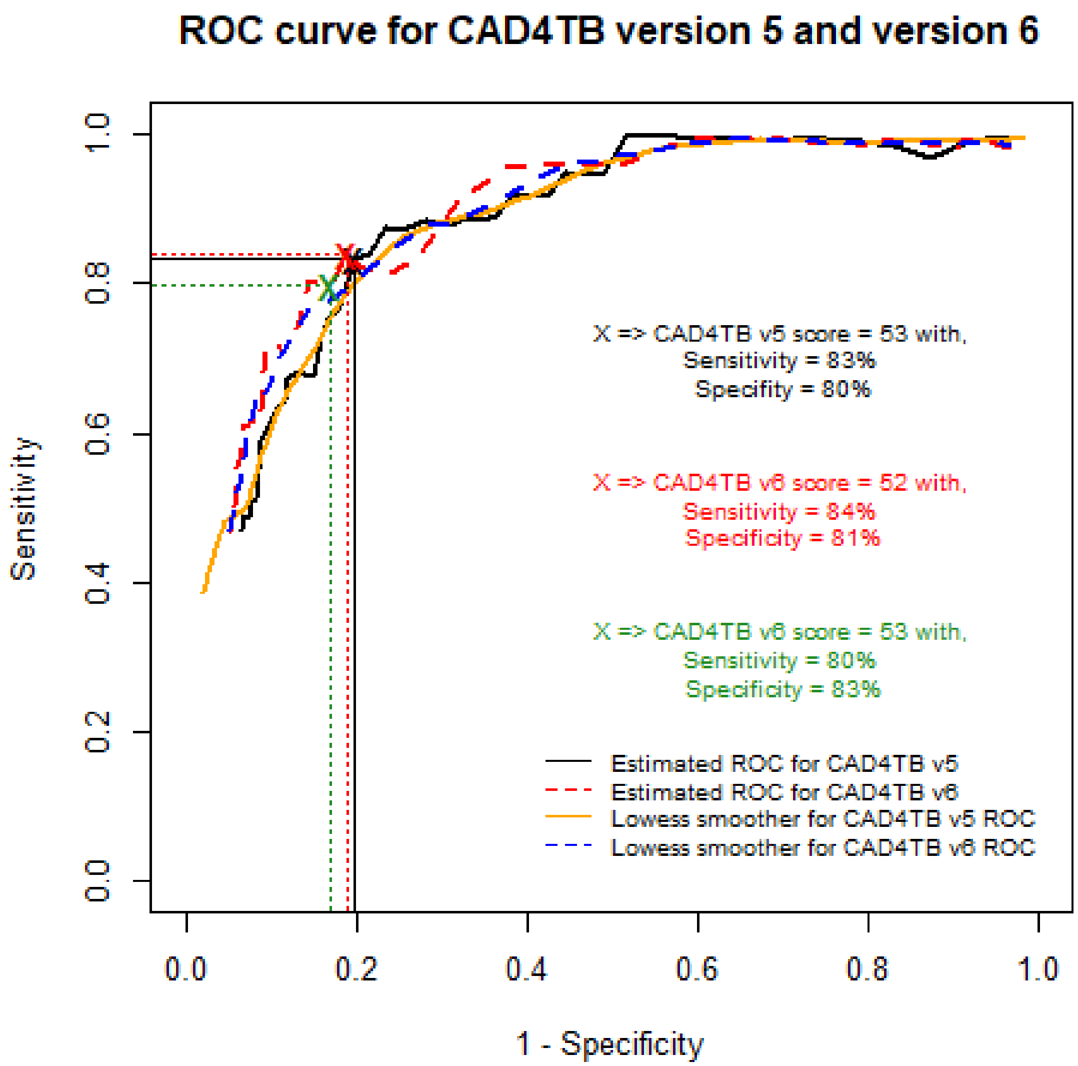

Fig S1: Receiver operating characteristic curves depicting the cut-off values used in the final latent class models

With these CAD4TB thresholds defined, we fitted a final model with any TB symptom, radiologist interpretation (chest X-ray abnormality suggestive of active TB), CAD4TBv5≥S_{t1}, CAD4TBv6≥S_{t2}, Xpert Ultra (excluding trace) and culture to estimate PTB prevalence, and diagnostic test sensitivity and specificity of the six diagnostic tests. We repeated the same analysis with chest X-ray abnormality suggestive of active TB replaced by any chest X-ray abnormality.

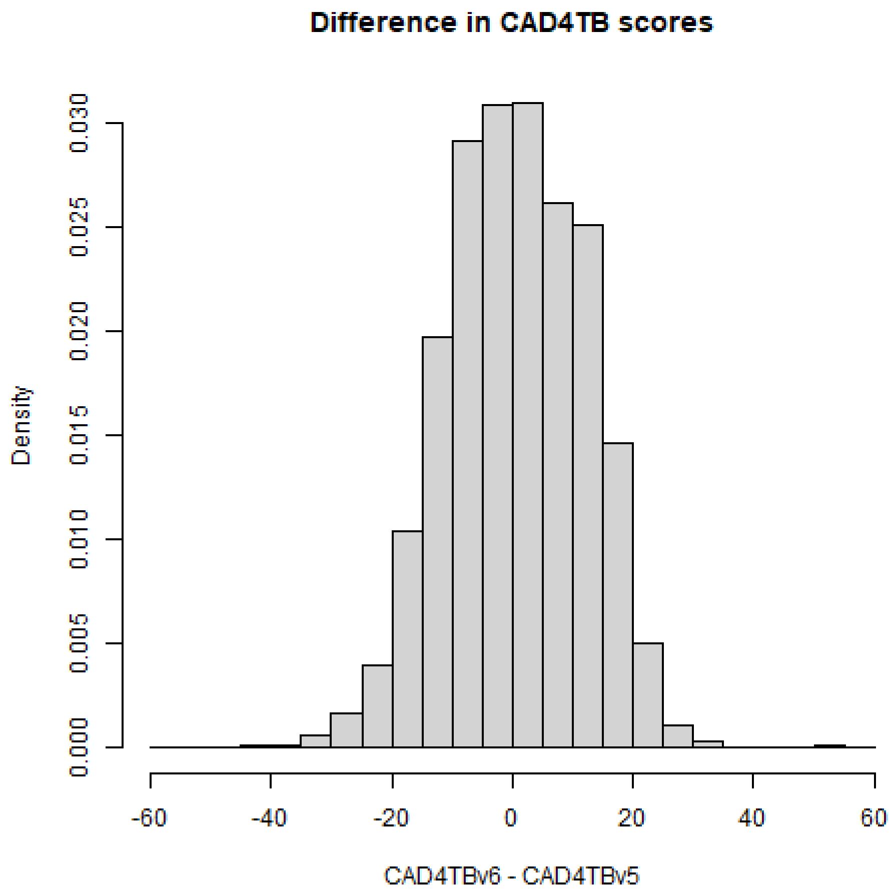

Fig S2: Distribution of difference in CAD4TB scores between version 6 and version 5

Fig S3 shows a scatter plot of CAD4TBv5 and CAD4TBv6 scores. The scatter plot reveals some trend in the scores. However, it is imperative to understand that the two devices are used to evaluate chest X-ray images of the same person. Therefore, they are paired. From the scatter plot, the positive correlation between version 6 and version 5 is not sufficiently strong to result in strong dependencies between the two versions in the model.

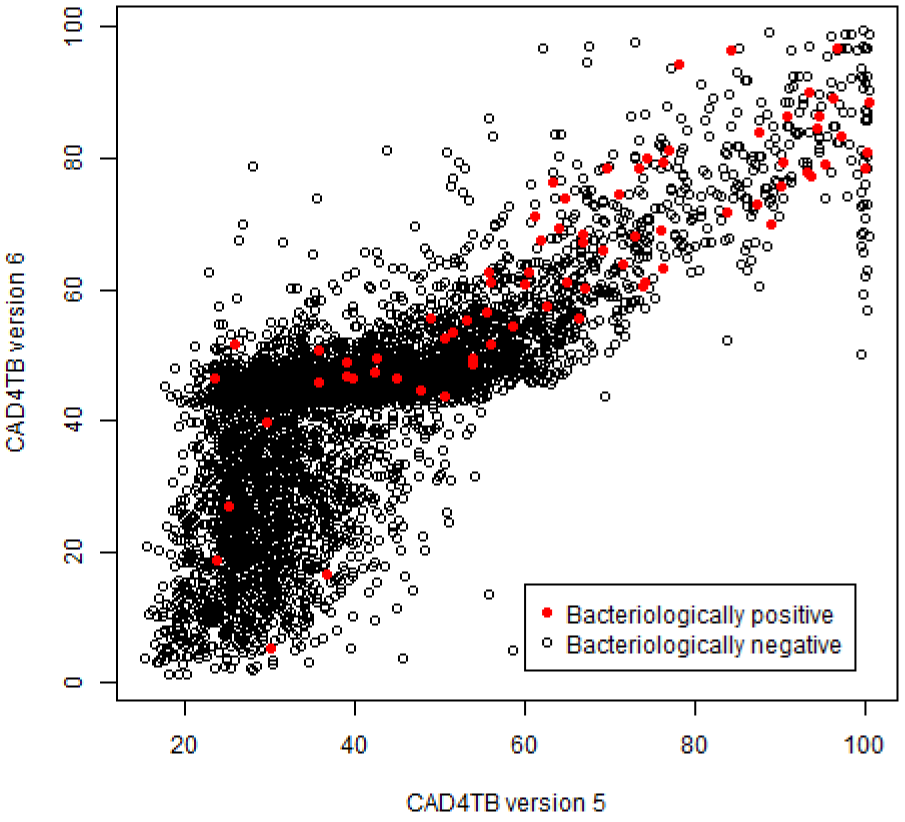

Fig S3: Scatter plot of CAD4TB version 5 and CAD4TB version 6 scores

4. Supplementary Tables

Table S7 presents results of four models. The results of Model 1, Model 2 and Model 3 were already presented in the main document but shown here to aid comparison with Model 4. These findings suggest that conditional dependence between TST and radiography among the children without CPTB cannot be ignored.

Table S6: Posterior median and 95% credible intervals (95% CrI) of the prevalence and diagnostic test accuracy of the parameters of childhood pulmonary tuberculosis dataset

		Model 0	Model 1	Model 2	Model 3	Model 4
**Test**	**Parameter**	**Median (95% CrI)**	**Median (95% CrI)**	**Median (95% CrI)**	**Median (95% CrI)**	**Median (95% CrI)**
	Prevalence	16.3 (13.6, 19.3)	21.6 (15.6, 28.6)	18.4 (14.7, 26.4)	18.9 (15.1, 27.0)	19.9 (15.4, 29.7)
TST	Sensitivity	69.2 (60.5, 76.9)	73.2 (61.8, 82.4)	68.3 (58.5, 79.9)	69.5 (59.4, 81.5)	70.5 (60.2, 83.0)
	Specificity	62.3 (58.4, 66.1)	65.2 (59.6, 71.8)	62.7 (58.5, 69.6)	63.1 (58.8, 70.4)	63.7 (59.2, 72.8)
Rad.	Sensitivity	66.0 (57.1, 74.1)	65.1 (56.1, 73.8)	64.5 (55.5, 73.3)	60.1 (42.5, 72.2)	62.7 (51.8, 76.8)
	Specificity	73.1 (69.5, 76.5)	74.8 (70.5, 79.2)	73.2 (69.4, 77.9)	72.2 (67.8, 76.5)	73.7 (69.4, 79.9)
SMM	Sensitivity	33.8 (25.5, 42.5)	24.3 (16.7, 34.8)	28.4 (18.7, 37.6)	28.4 (19.0, 37.2)	26.7 (16.7, 36.4)
	Specificity	99.9 (99.5, 100)	99.9 (99.5, 100)	99.9 (99.5, 100)	99.9 (99.5, 100)	99.9 (99.5, 100)
Xpert	Sensitivity	74.8 (66.3, 82.5)	59.1 (44.2, 75.7)	69.4 (47.8, 78.4)	70.3 (47.9, 79.5)	66.3 (43.2, 79.1)
	Specificity	98.2 (97.0, 99.3)	98.7 (97.1, 99.9)	98.7 (97.2, 99.9)	99.2 (97.5, 100)	99.0 (97.2, 100)
Culture	Sensitivity	99.9 (91.0, 100)	75.3 (56.9, 97.2)	88.0 (61.8, 98.4)	86.4 (60.3, 98.5)	82.7 (55.1, 98.2)
	Specificity	99.8 (99.0, 100)	99.8 (99.2, 100)	99.8 (99.2, 100)	99.8 (99.2, 100)	99.8 (99.2, 100)
	Deviance	183.7	104.7	102.1	101.7	107.8
	RMSE	42.4	38.0	12.0	9.1	32.9

Model 0 – Based on the assumption of conditional independence
Model 1 – Based on the expert opinion as detailed in [3]. The model accounts for conditional dependence between all the diagnostic tests except radiography among children with CPTB and conditional independence between all the diagnostic tests among children without CPTB
Model 2 – The model accounts for conditional dependence between all the diagnostic tests except radiography among children with CPTB and conditional dependence between TST and radiography among children without CPTB
Model 3 – Accounts for conditional dependence between all the diagnostic tests among children with CPTB and conditional dependence between TST and radiography among children without CPTB
Model 4 – Accounts for conditional dependence between all the diagnostic tests among children with CPTB and allows conditional independence between all the diagnostic tests among children without CPTB
TST – Tuberculin skin test, Rad. – Radiography, SMM – Sputum smear microscopy, Xpert – Xpert MTB/RIF
RMSE – Root mean squared error deviations. This is calculated as the square root of the sum of squared differences between the observed frequencies and the predicted frequencies. It shows how good the model is in explaining the variability in the data
Note model 3 seems to do better prediction compared to model 1 although model 1 seems to best fit the data. This may be explained by the fact that model 3 accounts for dependence between radiography and other diagnostic tests among the true CPTB cases. Hence extra penalty
CrI – Credible Intervals

Table S7: Distribution of the number of participants included in LCA by age, sex and HIV status

		Age (Years)				
		15 – 29 years	30 – 49 years	50 – 69 years	≥ 70 years	Total
Male	HIV +	56 (8.9%)	235 (48.4%)	148 (29.0%)	18 (9.6%)	457 (25.2%)
	HIV -	573 (91.1%)	251 (51.6%)	363 (71.0%)	169 (90.4%)	1356 (74.8%)
	Total	629 (34.7%)	486 (26.8%)	511 (28.2%)	187 (10.3%)	1813 (100%)
Female	HIV +	140 (26.6%)	507 (63.0%)	355 (29.3%)	37 (6.1%)	1039 (33.0%)
	HIV -	387 (73.4%)	298 (37.0%)	857 (70.7%)	566 (93.9%)	2108 (67.0%)
	Total	527 (16.7%)	805 (25.6%)	1212 (38.5%)	603 (19.2%)	3147 (100%)
Total	HIV +	196 (17.0%)	742 (57.5%)	503 (29.2%)	55 (7.0%)	1496 (30.2%)
	HIV -	960 (83.0%)	549 (42.5%)	1220 (70.8%)	735 (93.0%)	3464 (69.8%)
	Total	1156 (23.3%)	1291 (26.0%)	1723 (34.7%)	790 (15.9%)	4960 (100%)

Table S8: Posterior median and 95% credible intervals (95% CrI) of the age, sex and HIV adjusted PTB prevalence and diagnostic test sensitivity and specificity among male individuals in Vukuzazi dataset

			**15 – 29 years**	**30 – 49 years**	**50 – 69 years**	**≥70 years**
**Group**	**Test**	**Parameter**	**Median (95% CrI)**	**Median (95% CrI)**	**Median (95% CrI)**	**Median (95% CrI)**
**HIV+ Male**		Prevalence	1.5 (0.8, 2.4)	1.6 (0.9, 2.7)	1.9 (1.1, 3.2)	1.6 (0.8, 3.0)
	Any TB symptom	Sensitivity	28.1 (12.0, 51.2)	22.2 (7.2, 46.0)	27.8 (11.0, 50.1)	26.7 (7.6, 55.3)
		Specificity	82.3 (78.8, 85.4)	84.4 (81.6, 87.0)	84.4 (81.3, 87.2)	83.9 (79.7, 87.6)
	Radiologist conclusion‡	Sensitivity	90.2 (72.9, 97.7)	90.8 (72.8, 98.3)	94.9 (82.0, 99.2)	93.2 (74.0, 99.1)
		Specificity	77.8 (74.1, 81.4)	54.8 (51.1, 58.6)	44.3 (40.2, 48.5)	37.2 (32.0, 42.6)
	CAD4TBv5≥53	Sensitivity	86.4 (67.3, 96.2)	88.2 (67.5, 97.6)	88.6 (70.1, 97.3)	88.6 (65.8, 97.9)
		Specificity	87.3 (84.0, 90.2)	68.7 (65.1, 72.4)	56.3 (51.7, 60.7)	37.5 (32.0, 43.3)
	CAD4TBv6≥53	Sensitivity	83.3 (63.7, 94.7)	83.7 (60.8, 96.0)	86.6 (66.8, 96.6)	85.3 (59.9, 96.9)
		Specificity	89.8 (86.7, 92.3)	69.9 (66.2, 73.5)	57.0 (52.5, 61.6)	41.2 (35.7, 47.0)
	Xpert Ultra†	Sensitivity	65.1 (40.6, 85.3)	70.1 (42.7, 89.5)	69.9 (45.5, 88.0)	62.5 (31.9, 87.1)
		Specificity	99.4 (99.1, 99.6)	99.4 (99.1, 99.6)	99.4 (99.1, 99.6)	99.4 (99.1, 99.6)
	Culture	Sensitivity	76.7 (52.0, 94.1)	76.7 (47.7, 95.8)	71.1 (44.8, 92.9)	81.4 (52.8, 96.8)
		Specificity	99.8 (99.7, 99.9)	99.8 (99.7, 99.9)	99.8 (99.7, 99.9)	99.8 (99.7, 99.9)
**HIV- Male**		Prevalence	0.9 (0.5, 1.4)	1.0 (0.6, 1.7)	1.2 (0.7, 2.0)	1.0 (0.5, 1.8)
	Any TB symptom	Sensitivity	26.1 (12.0, 44.6)	20.2 (6.4, 42.3)	25.5 (10.5, 47.1)	24.8 (7.9, 49.7)
		Specificity	81.9 (79.2, 84.3)	84.0 (81.1, 86.6)	84.0 (81.6, 86.3)	83.5 (80.1, 86.3)
	Radiologist conclusion‡	Sensitivity	83.4 (66.8, 93.8)	84.2 (63.2, 95.8)	90.5 (74.7, 97.7)	88.1 (67.3, 97.3)
		Specificity	85.9 (83.6, 88.1)	66.6 (63.0, 70.1)	56.6 (53.3, 60.0)	49.3 (45.0, 53.6)
	CAD4TBv5≥53	Sensitivity	82.5 (64.9, 93.7)	84.8 (63.0, 96.2)	85.5 (65.4, 96.1)	85.2 (63.2, 96.6)
		Specificity	91.2 (89.2, 93.1)	76.2 (72.7, 79.5)	64.8 (61.4, 68.2)	46.2 (41.7, 50.7)
	CAD4TBv6≥53	Sensitivity	80.8 (62.5, 92.7)	81.5 (58.3, 94.7)	84.8 (65.2, 95.6)	83.2 (60.0, 95.7)
		Specificity	94.4 (92.8, 95.8)	80.0 (76.7, 83.0)	69.0 (65.6, 72.2)	53.9 (49.4, 58.3)
	Xpert Ultra†	Sensitivity	69.3 (48.8, 85.3)	73.7 (49.0, 90.7)	73.5 (51.5, 89.6)	66.8 (39.2, 87.8)
		Specificity	99.4 (99.1, 99.6)	99.4 (99.1, 99.6)	99.4 (99.1, 99.6)	99.4 (99.1, 99.6)
	Culture	Sensitivity	78.2 (58.4, 92.9)	78.1 (51.6, 95.2)	72.6 (48.6, 92.5)	82.6 (58.1, 96.2)
		Specificity	99.8 (99.7, 99.9)	99.8 (99.7, 99.9)	99.8 (99.7, 99.9)	99.8 (99.7, 99.9)

‡ - Any chest X-ray abnormality, † - Excluding trace

Table S9: Posterior median and 95% credible intervals (95% CrI) of the age, sex and HIV adjusted PTB prevalence and diagnostic test sensitivity and specificity among female individuals in Vukuzazi dataset

			15 – 29 years	30 – 49 years	50 – 69 years	≥70 years
**Group**	**Test**	**Parameter**	**Median (95% CrI)**	**Median (95% CrI)**	**Median (95% CrI)**	**Median (95% CrI)**
**HIV+ Female**		Prevalence	0.9 (0.5, 1.5)	1.0 (0.5, 1.7)	1.2 (0.6, 2.0)	1.0 (0.5, 1.8)
	Any TB	Sensitivity	30.2 (14.8, 51.4)	24.1 (9.1, 46.8)	29.8 (12.5, 52.3)	28.9 (9.5, 55.7)
	symptom	Specificity	81.4 (77.8, 84.5)	83.6 (81.1, 85.9)	83.6 (80.9, 85.9)	83.0 (79.3, 86.4)
	Radiologist	Sensitivity	84.9 (67.3, 94.8)	85.7 (65.7, 96.3)	91.6 (74.8, 98.2)	89.2 (67.6, 97.9)
	conclusion‡	Specificity	84.2 (81.1, 86.9)	63.8 (60.7, 66.9)	53.6 (50.2, 57.1)	46.3 (41.5, 51.0)
	CAD4TBv5≥53	Sensitivity	79.5 (60.4, 92.4)	81.8 (59.8, 95.3)	82.6 (61.5, 94.8)	82.4 (57.6, 95.8)
		Specificity	96.1 (94.7, 97.3)	86.8 (84.5, 88.9)	78.4 (75.4, 81.2)	62.1 (57.1, 66.9)
	CAD4TBv6≥53	Sensitivity	80.8 (61.9, 92.7)	81.5 (59.2, 94.7)	84.7 (65.1, 95.4)	83.2 (58.1, 95.8)
		Specificity	96.8 (95.5, 97.9)	86.9 (84.7, 89.0)	78.3 (75.3, 81.2)	64.8 (59.9, 69.5)
	Xpert Ultra†	Sensitivity	52.2 (31.4, 72.4)	57.4 (33.2, 79.8)	57.3 (32.8, 78.7)	49.3 (23.5, 76.3)
		Specificity	99.4 (99.1, 99.6)	99.4 (99.1, 99.6)	99.4 (99.1, 99.6)	99.4 (99.1, 99.6)
	Culture	Sensitivity	76.9 (55.5, 92.6)	77.1 (50.6, 94.5)	71.4 (45.6, 92.0)	81.5 (55.5, 96.1)
		Specificity	99.8 (99.7, 99.9)	99.8 (99.7, 99.9)	99.8 (99.7, 99.9)	99.8 (99.7, 99.9)
**HIV- Female**		Prevalence	0.5 (0.3, 0.9)	0.6 (0.3, 1.0)	0.7 (0.4, 1.2)	0.6 (0.3, 1.1)
	Any TB	Sensitivity	28.1 (15.4, 44.0)	22.2 (8.2, 43.4)	27.4 (11.9, 49.1)	26.6 (9.9, 50.5)
	symptom	Specificity	80.9 (78.1, 83.6)	83.2 (80.3, 85.8)	83.1 (81.0, 85.1)	82.6 (79.8, 85.2)
	Radiologist	Sensitivity	76.4 (60.8, 87.8)	77.5 (54.9, 91.6)	85.5 (66.8, 95.5)	82.1 (59.7, 94.7)
	conclusion‡	Specificity	90.5 (88.6, 92.2)	74.6 (71.4, 77.7)	65.6 (62.9, 68.1)	58.5 (54.8, 62.0)
	CAD4TBv5≥53	Sensitivity	74.6 (57.5, 87.3)	77.4 (54.7, 92.5)	78.5 (56.6, 92.5)	78.1 (55.0, 93.0)
		Specificity	97.6 (96.8, 98.3)	91.0 (89.0, 92.7)	84.3 (82.3, 86.2)	70.2 (66.9, 73.3)
	CAD4TBv6≥53	Sensitivity	77.9 (61.8, 89.8)	78.8 (56.4, 93.0)	82.4 (62.4, 94.1)	80.7 (58.3, 94.0)
		Specificity	98.5 (97.9, 99.0)	92.5 (90.8, 94.1)	86.4 (84.5, 88.2)	75.7 (72.7, 78.5)
	Xpert Ultra†	Sensitivity	56.5 (40.0, 72.6)	61.9 (37.6, 82.3)	61.4 (38.5, 81.6)	53.7 (30.1, 77.2)
		Specificity	99.4 (99.1, 99.6)	99.4 (99.1, 99.6)	99.4 (99.1, 99.6)	99.4 (99.1, 99.6)
	Culture	Sensitivity	78.5 (61.9, 90.6)	78.5 (54.7, 93.8)	73.0 (48.8, 91.7)	82.8 (60.8, 95.2)
		Specificity	99.8 (99.7, 99.9)	99.8 (99.7, 99.9)	99.8 (99.7, 99.9)	99.8 (99.7, 99.9)

‡ - Any chest X-ray abnormality, † - Excluding trace

5. Supplementary Figures

Model Diagnostics

Trace plots for assessing mixing in the chains

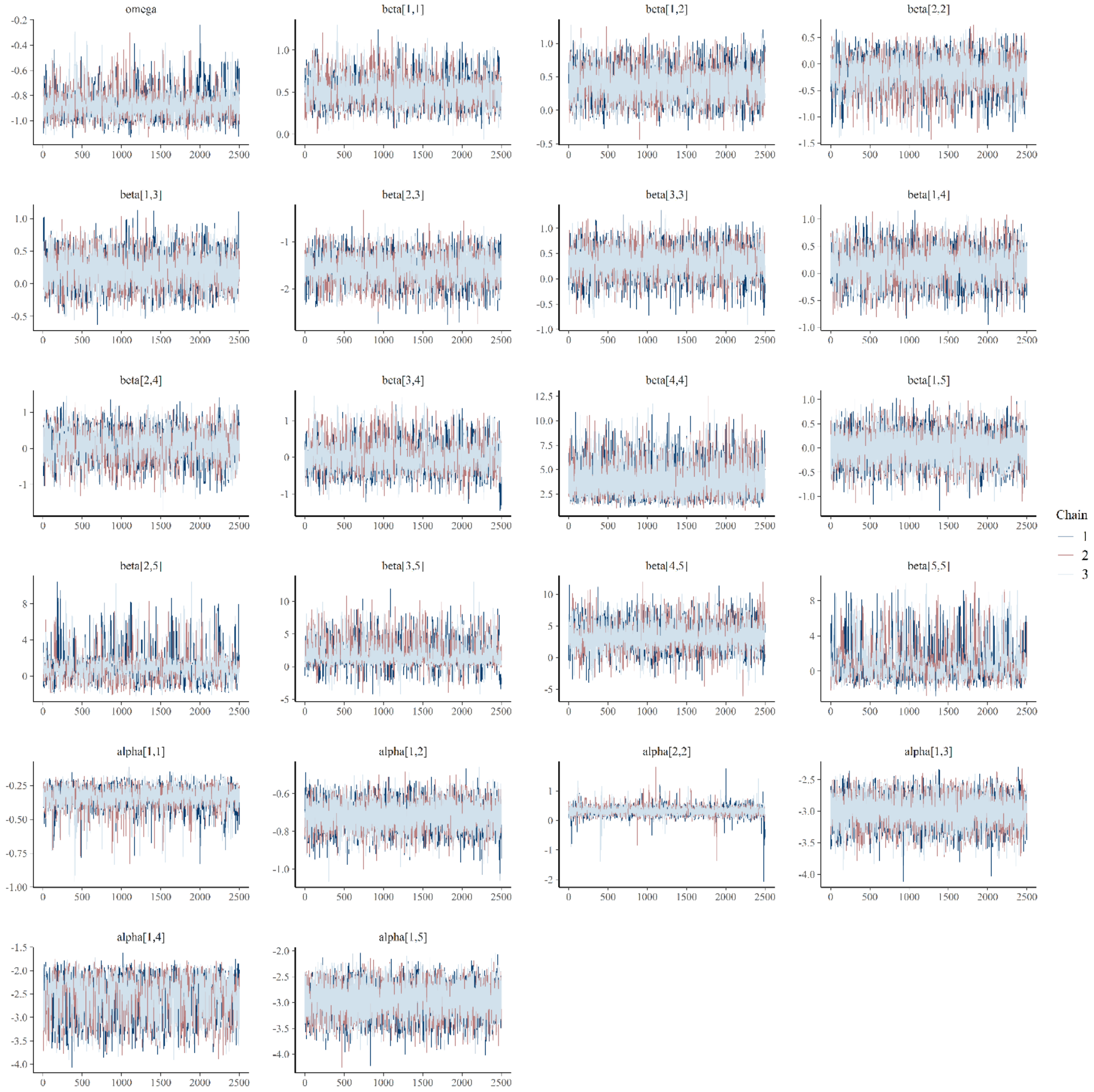

Fig S4: Trace plots of parameters on the probit scale corresponding to prevalence (ω) and parameters of the probit regression models for conditional probabilities of diagnostic tests in the childhood pulmonary TB data analyzed using Model 2 presented in Table S1

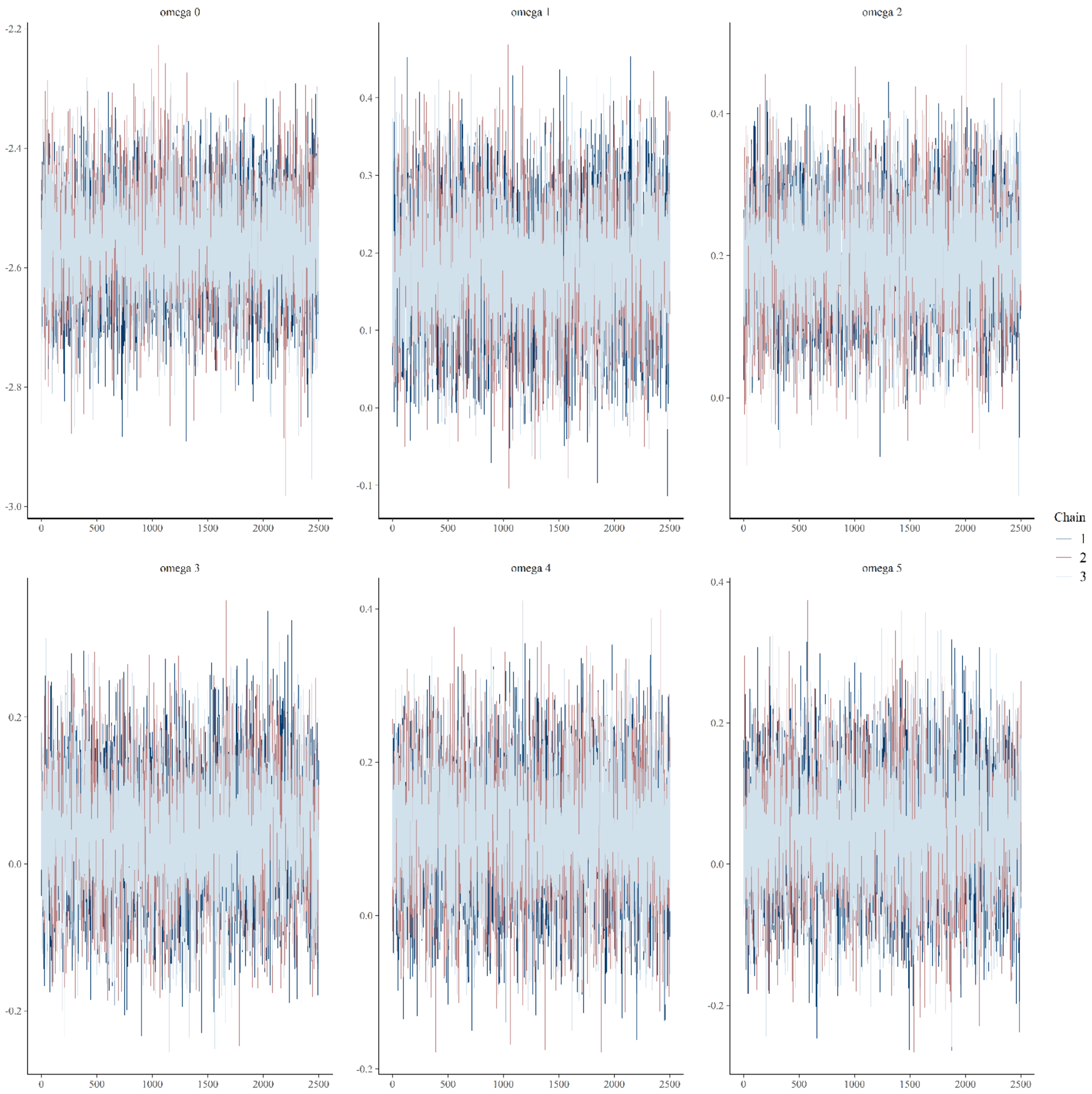

Fig S5: Trace plots of parameters of probit regression model for pulmonary TB prevalence in Vukuzazi dataset analyzed using Model 2 presented in Table S4

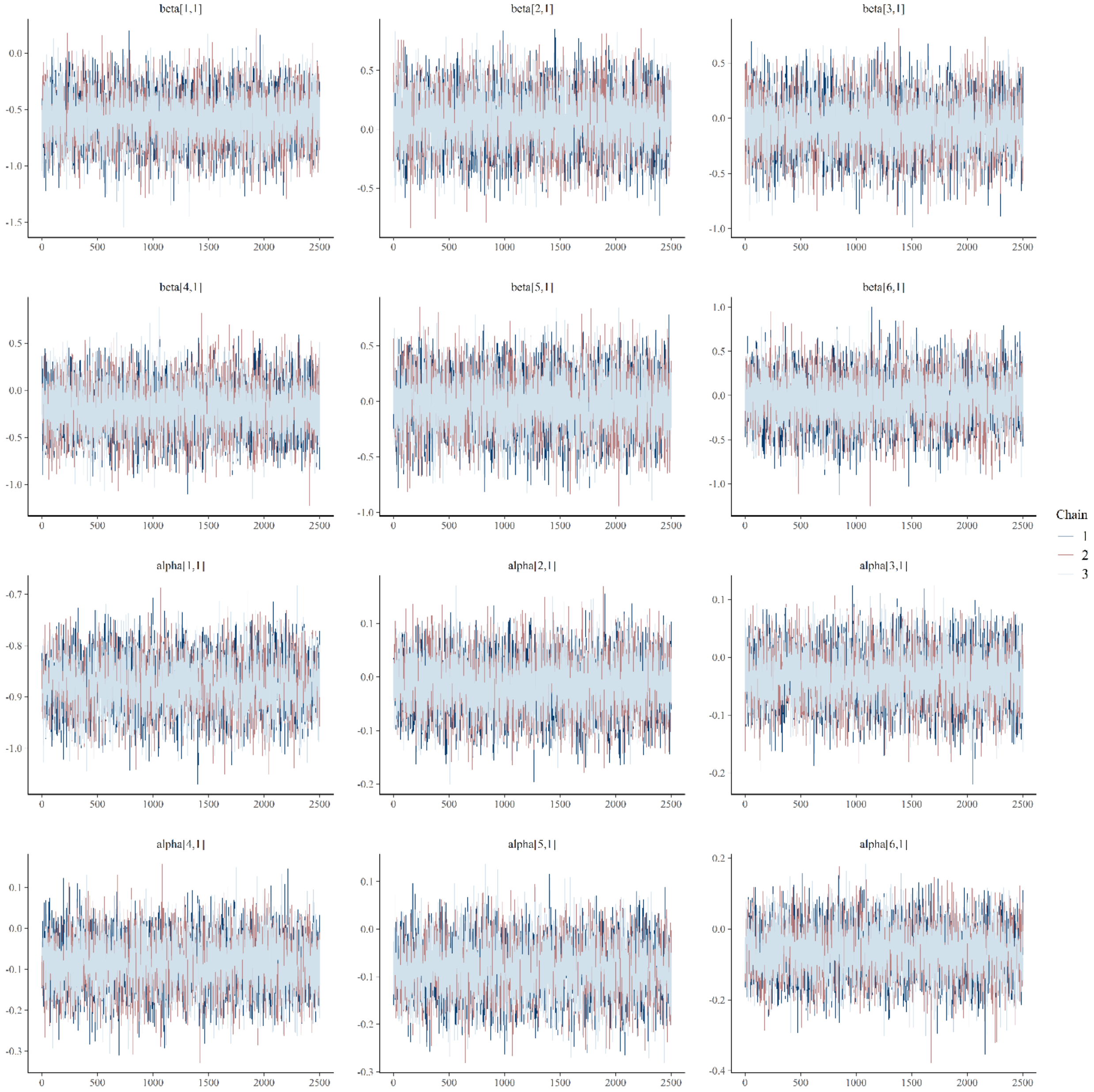

Fig S6: Trace plots of parameters of the probit regression model for conditional probability of any TB symptom among the true PTB cases (β) and among the true non-PTB cases (α) in the Vukuzazi dataset analyzed using Model 2 presented in Table S4

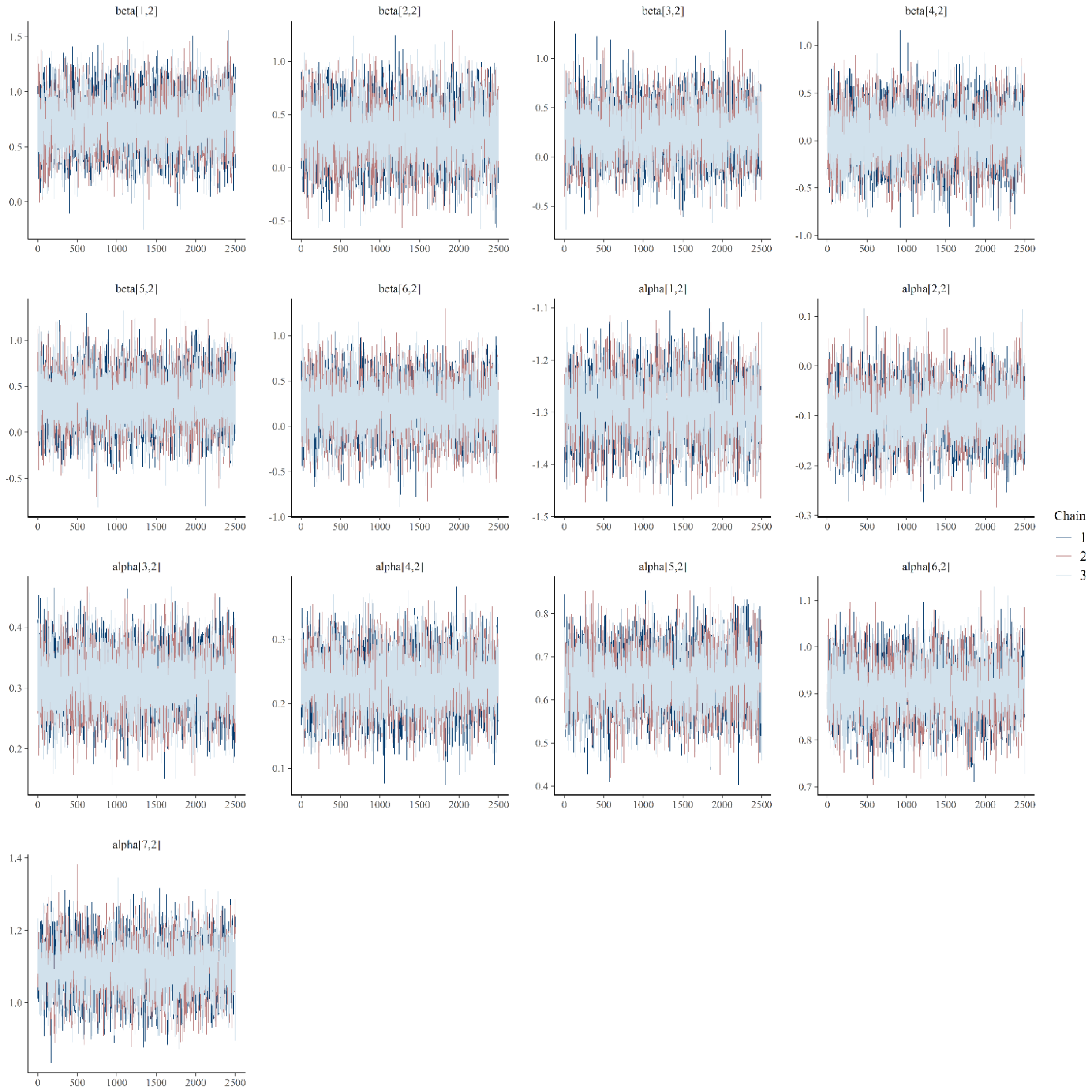

Fig S7: Trace plots of parameters of the probit regression model for conditional probability of radiologist conclusion (any chest X-ray abnormality) among the true PTB cases (β) and among the true non-PTB cases (α) in the Vukuzazi dataset analyzed using Model 2 presented in Table S4

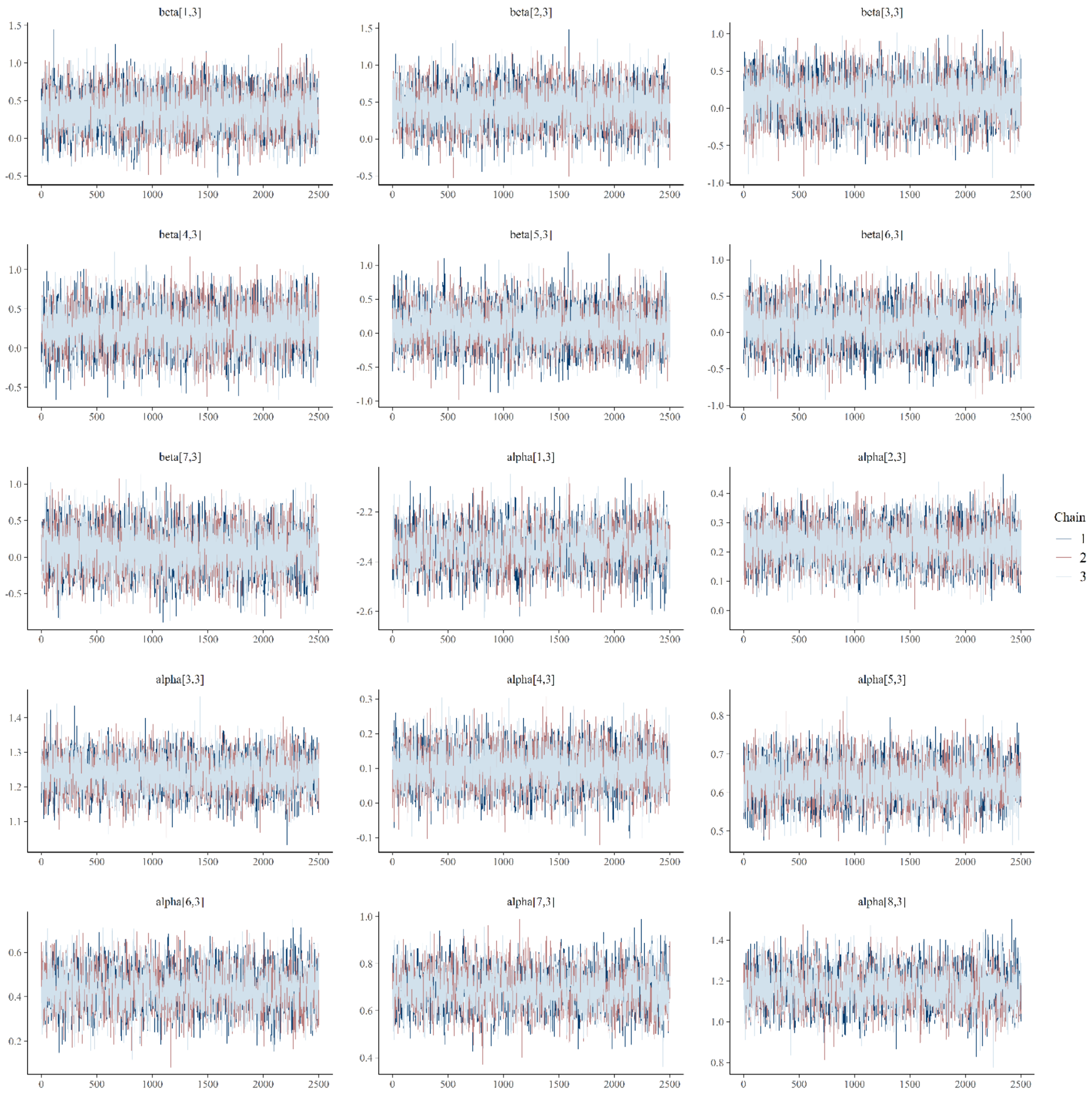

Fig S8: Trace plots of parameters of the probit regression model for conditional probability of CAD4TBv5≥53 among the true PTB cases (β) and among the true non-PTB cases (α) in the Vukuzazi dataset analyzed using Model 2 presented in Table S4

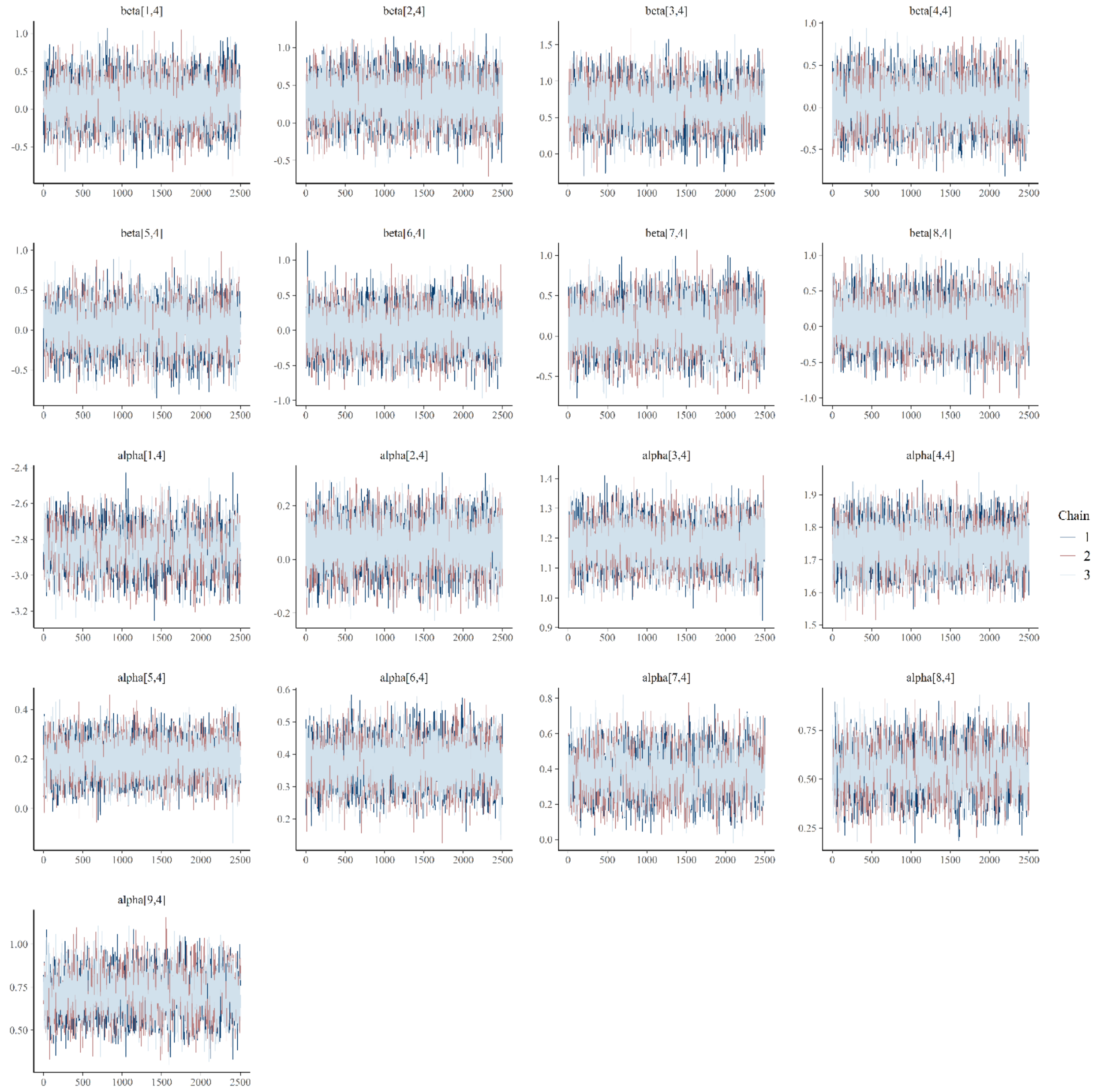

Fig S9: Trace plots of parameters of the probit regression model for conditional probability of CAD4TBv6≥53 among the true PTB cases (β) and among the true non-PTB cases (α) in the Vukuzazi dataset analyzed using Model 2 presented in Table S4

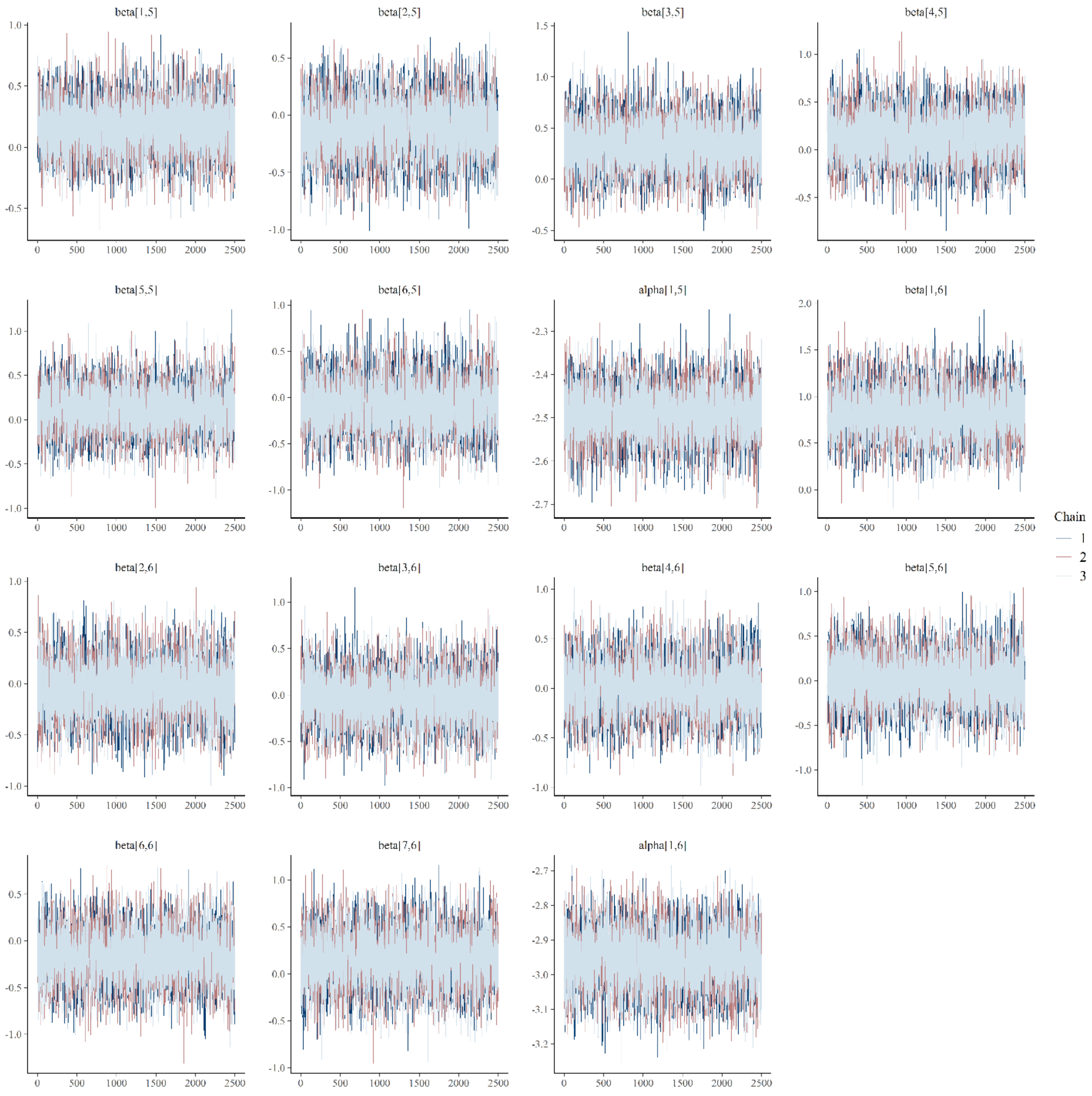

Fig S10: Trace plots of parameters of the probit regression model for conditional probability of Xpert Ultra and culture among the true PTB cases (β) and among the true non-PTB cases (α) in the Vukuzazi dataset analyzed using Model 2 presented in Table S4

6. Analysis scripts

Below is the Rjags script for model 2 used for analyzing childhood pulmonary TB data. The other models can be adapted from this model.

```
model2 <- function(){
#K is the number of columns in y
#The first column of y is 1s
#We have K-1 tests
  ## likelihood -----------------------------------------------------------------------------------------
  Freqobs[1:J] ~ dmulti(p[1:J],N)
  for (j in 1:J) {      # Prob. of a combination of J test results
    p[j] <- pi*prod(cp1[j,1:(K-1)]^y[j,2:K]*(1-cp1[j,1:(K-1)])^(1-y[j,2:K])) +
      (1-pi)*prod(cp0[j,1:(K-1)]^(y[j,2:K])*(1-cp0[j,1:(K-1)])^(1-y[j,2:K]))

      cp1[j,1]  =  phi( inprod( y[j,c(1:1)], beta[1:1,1] ) )  #Modeling pr(y_1 = + | D=+)
      cp1[j,2]  =  phi( inprod( y[j,c(1:2)], beta[1:2,2] ) )  #Modeling pr(y_2 = + | y_1, D=+)
      cp1[j,3]  =  phi( inprod( y[j,c(1:3)], beta[1:3,3] ) )  #Modeling pr(y_3 = + | y_1, y_2, D=+)
      cp1[j,4]  =  phi( inprod( y[j,c(1:4)], beta[1:4,4] ) )  #Modeling pr(y_4 = + | y_1, y_2, y_3, D=+)
      cp1[j,5]  =  phi( inprod( y[j,c(1:5)], beta[1:5,5] ) )  #Modeling pr(y_5 = + | y_1, y_2, y_3, y_4, D=+)

       #Predict conditional probabilities
      jp1x[j,1] = y[j,2]*cp1[j,1] + (1-y[j,2])*(1-cp1[j,1])
      jp1x[j,2] = y[j,3]*cp1[j,2] + (1-y[j,3])*(1-cp1[j,2])
      jp1x[j,3] = y[j,4]*cp1[j,3] + (1-y[j,4])*(1-cp1[j,3])
      jp1x[j,4] = y[j,5]*cp1[j,4] + (1-y[j,5])*(1-cp1[j,4])
      jp1x[j,5] = y[j,6]*cp1[j,5] + (1-y[j,6])*(1-cp1[j,5])

      cp0[j,1]  =  phi( inprod( y[j,c(1:1)], alpha[1:1,1] ) )  # Modeling pr(y_1 = + | D=-)
      cp0[j,2]  =  phi( inprod( y[j,c(1:2)], alpha[1:2,2] ) )  # Modeling pr(y_2 = + | y_1, D=-)
      cp0[j,3]  =  phi( inprod( y[j,c(1:1)], alpha[1:1,3] ) )  # Modeling pr(y_3 = + | y_1, y_2, D=-)
      cp0[j,4]  =  phi( inprod( y[j,c(1:1)], alpha[1:1,4] ) )  # Modeling pr(y_4 = + | y_1, y_2, y_3, D=-)
      cp0[j,5]  =  phi( inprod( y[j,c(1:1)], alpha[1:1,5] ) )  #Modeling pr(y_5 = + | y_1, y_2, y_3, y_4, D=-)

       #Predict conditional probabilities
      jp0x[j,1] = y[j,2]*cp0[j,1] + (1-y[j,2])*(1 - cp0[j,1])
      jp0x[j,2] = y[j,3]*cp0[j,2] + (1-y[j,3])*(1 - cp0[j,2])
      jp0x[j,3] = y[j,4]*cp0[j,3] + (1-y[j,4])*(1 - cp0[j,3])
      jp0x[j,4] = y[j,5]*cp0[j,4] + (1-y[j,5])*(1 - cp0[j,4])
      jp0x[j,5] = y[j,6]*cp0[j,5] + (1-y[j,6])*(1 - cp0[j,5])

    jp1[j,1] = prod( jp1x[j,1] )
    jp1[j,2] = prod( jp1x[j,1:2] )
    jp1[j,3] = prod( jp1x[j,1:3] )
    jp1[j,4] = prod( jp1x[j,1:4] )
    jp1[j,5] = prod( jp1x[j,1:5] )

    jp0[j,1] = prod( jp0x[j,1] )
    jp0[j,2] = prod( jp0x[j,1:2] )
    jp0[j,3] = prod( jp0x[j,3] )
    jp0[j,4] = prod( jp0x[j,4] )
    jp0[j,5] = prod( jp0x[j,5] )
```

```
  }
#calculate the sensitivity and specificity
    se[1] =     sum( jp1[c(1:1), 1] )
    se[2] =     sum( jp1[c(1:2), 2] )
    se[3] =     sum( jp1[c(1:4), 3] )
    se[4] =     sum( jp1[c(1:8), 4] )
    se[5] =     sum( jp1[c(1:16), 5] )

    sp[1] = 1 - sum( jp0[c(1:1), 1] )
    sp[2] = 1 - sum( jp0[c(1:2), 2] )
    sp[3] = 1 - sum( jp0[c(1:1), 3] )
    sp[4] = 1 - sum( jp0[c(1:1), 4] )
    sp[5] = 1 - sum( jp0[c(1:1), 5] )

  pi <- phi(omega)

  ## priors ------------------------------------------------------------------------------------------
for(g in 1:(K-1)){
  for(r in 1:g1[g]){
    beta[r,g] ~ dnorm(mu1[r,g], s1[r,g])
  }
}
  for(g in 1:(K-1)){
    for(r in 1:g0[g]){
      alpha[r,g] ~ dnorm(mu0[r,g], s0[r,g])
    }
  }
  # Prior for prevalence
  omega ~ dnorm( mu.prev, s.prev )
}

#Choice of priors (Determined outside jags and issued as part of the data)
g1 = c(1,2,3,4,5)  # Number of parameters in models for CPTB cases
g0 = c(1,2,1,1,1)  # Number of parameters in models for non-CPTB cases

mu1 = matrix(NA, nrow = max(g1), ncol = K)  #Mean among CPTB cases
s1 = matrix(NA, nrow = max(g1), ncol = K)  # Precision for mean among the CPTB cases

for(c in 1:ncol(mu1)){
  for(r in 1:c){
  mu1[r,c] = 0
  }
}

for(c in 1:ncol(s1)){
  for(r in 1:c){
    s1[r,c] = 0.1
  }
}
for(c in 1:ncol(s1)){
  for(r in 1:1){
    s1[r,c] = 1
  }
```

```
}

s1[1,c(3,4,5)] <- 10

mu0 = matrix(NA, nrow = max(g0), ncol = K)  #Mean among non CPTB cases
s0 = matrix(NA, nrow = max(g0), ncol = K)  # Precision for mean among the non CPTB cases

for(c in 1:ncol(mu0)){
  for(r in 1:2){
    mu0[r,c] = 0
  }
}
mu0[1,3:5] <- -3

for(c in 1:ncol(s0)){
  for(r in 1:2){
    s0[r,c] = 1
  }
}
for(c in 1:ncol(s0)){
  for(r in 1:1){
    s0[r,c] = 10
  }
}
#mean and precision for the prior distribution of CPTB prevalence
mu.prev = 0
s.prev = 1
```

Below is the Rjags script for model 2 used for analyzing Vukuzazi data. The other models can be adapted from this model.

```
cond.prob.jags.model1_11_2 <- function(){
#K is the number of columns in y
#The first column of y is 1s
#We have K-1 tests
  ## likelihood ------------------------------------------------------------------------------------------
  Freqobs[1:J] ~ dmulti(p[1:J],N)
  for (j in 1:J) {    # Prob. of test j yielding +ve result given disease status
    p[j] <- ( pa[1]*(psij[j]*((gammaj[j]*(pij[j]*prod(cp1[j,1:(K-1)]^y[j,2:K]*(1-cp1[j,1:(K-1)])^(1-y[j,2:K])) +
                                   (1-pij[j])*prod(cp0[j,1:(K-1)]^(y[j,2:K])*(1-cp0[j,1:(K-1)])^(1-y[j,2:K]))))^y[j,8]*
                           ((1-gammaj[j])*(pij[j]*prod(cp1[j,1:(K-1)]^y[j,2:K]*(1-cp1[j,1:(K-1)])^(1-y[j,2:K])) +
                                      (1-pij[j])*prod(cp0[j,1:(K-1)]^(y[j,2:K])*(1-cp0[j,1:(K-1)])^(1-y[j,2:K]))))^(1-
y[j,8])))^y[j,9]*
              ((1-psij[j])*((gammaj[j]*(pij[j]*prod(cp1[j,1:(K-1)]^y[j,2:K]*(1-cp1[j,1:(K-1)])^(1-y[j,2:K])) +
                                   (1-pij[j])*prod(cp0[j,1:(K-1)]^(y[j,2:K])*(1-cp0[j,1:(K-1)])^(1-y[j,2:K]))))^y[j,8]*
                           ((1-gammaj[j])*(pij[j]*prod(cp1[j,1:(K-1)]^y[j,2:K]*(1-cp1[j,1:(K-1)])^(1-y[j,2:K])) +
                                      (1-pij[j])*prod(cp0[j,1:(K-1)]^(y[j,2:K])*(1-cp0[j,1:(K-1)])^(1-y[j,2:K]))))^(1-y[j,8])))^(1-
y[j,9]) )^((y[j,10]==1)*1)*
      ( pa[2]*(psij[j]*((gammaj[j]*(pij[j]*prod(cp1[j,1:(K-1)]^y[j,2:K]*(1-cp1[j,1:(K-1)])^(1-y[j,2:K])) +
                                (1-pij[j])*prod(cp0[j,1:(K-1)]^(y[j,2:K])*(1-cp0[j,1:(K-1)])^(1-y[j,2:K]))))^y[j,8]*
                        ((1-gammaj[j])*(pij[j]*prod(cp1[j,1:(K-1)]^y[j,2:K]*(1-cp1[j,1:(K-1)])^(1-y[j,2:K])) +
                                   (1-pij[j])*prod(cp0[j,1:(K-1)]^(y[j,2:K])*(1-cp0[j,1:(K-1)])^(1-y[j,2:K]))))^(1-y[j,8])))^y[j,9]*
        ((1-psij[j])*((gammaj[j]*(pij[j]*prod(cp1[j,1:(K-1)]^y[j,2:K]*(1-cp1[j,1:(K-1)])^(1-y[j,2:K])) +
                                (1-pij[j])*prod(cp0[j,1:(K-1)]^(y[j,2:K])*(1-cp0[j,1:(K-1)])^(1-y[j,2:K]))))^y[j,8]*
                        ((1-gammaj[j])*(pij[j]*prod(cp1[j,1:(K-1)]^y[j,2:K]*(1-cp1[j,1:(K-1)])^(1-y[j,2:K])) +
                                   (1-pij[j])*prod(cp0[j,1:(K-1)]^(y[j,2:K])*(1-cp0[j,1:(K-1)])^(1-y[j,2:K]))))^(1-y[j,8])))^(1-y[j,9])
)^((y[j,10]==2)*1)*
      ( pa[3]*(psij[j]*((gammaj[j]*(pij[j]*prod(cp1[j,1:(K-1)]^y[j,2:K]*(1-cp1[j,1:(K-1)])^(1-y[j,2:K])) +
                                (1-pij[j])*prod(cp0[j,1:(K-1)]^(y[j,2:K])*(1-cp0[j,1:(K-1)])^(1-y[j,2:K]))))^y[j,8]*
                        ((1-gammaj[j])*(pij[j]*prod(cp1[j,1:(K-1)]^y[j,2:K]*(1-cp1[j,1:(K-1)])^(1-y[j,2:K])) +
                                   (1-pij[j])*prod(cp0[j,1:(K-1)]^(y[j,2:K])*(1-cp0[j,1:(K-1)])^(1-y[j,2:K]))))^(1-y[j,8])))^y[j,9]*
        ((1-psij[j])*((gammaj[j]*(pij[j]*prod(cp1[j,1:(K-1)]^y[j,2:K]*(1-cp1[j,1:(K-1)])^(1-y[j,2:K])) +
                                (1-pij[j])*prod(cp0[j,1:(K-1)]^(y[j,2:K])*(1-cp0[j,1:(K-1)])^(1-y[j,2:K]))))^y[j,8]*
                        ((1-gammaj[j])*(pij[j]*prod(cp1[j,1:(K-1)]^y[j,2:K]*(1-cp1[j,1:(K-1)])^(1-y[j,2:K])) +
                                   (1-pij[j])*prod(cp0[j,1:(K-1)]^(y[j,2:K])*(1-cp0[j,1:(K-1)])^(1-y[j,2:K]))))^(1-y[j,8])))^(1-y[j,9])
)^((y[j,10]==3)*1)*
      ( (pa[4])*(psij[j]*((gammaj[j]*(pij[j]*prod(cp1[j,1:(K-1)]^y[j,2:K]*(1-cp1[j,1:(K-1)])^(1-y[j,2:K])) +
                                  (1-pij[j])*prod(cp0[j,1:(K-1)]^(y[j,2:K])*(1-cp0[j,1:(K-1)])^(1-y[j,2:K]))))^y[j,8]*
                          ((1-gammaj[j])*(pij[j]*prod(cp1[j,1:(K-1)]^y[j,2:K]*(1-cp1[j,1:(K-1)])^(1-y[j,2:K])) +
                                     (1-pij[j])*prod(cp0[j,1:(K-1)]^(y[j,2:K])*(1-cp0[j,1:(K-1)])^(1-y[j,2:K]))))^(1-y[j,8])))^y[j,9]*
        ((1-psij[j])*((gammaj[j]*(pij[j]*prod(cp1[j,1:(K-1)]^y[j,2:K]*(1-cp1[j,1:(K-1)])^(1-y[j,2:K])) +
                                (1-pij[j])*prod(cp0[j,1:(K-1)]^(y[j,2:K])*(1-cp0[j,1:(K-1)])^(1-y[j,2:K]))))^y[j,8]*
                        ((1-gammaj[j])*(pij[j]*prod(cp1[j,1:(K-1)]^y[j,2:K]*(1-cp1[j,1:(K-1)])^(1-y[j,2:K])) +
                                   (1-pij[j])*prod(cp0[j,1:(K-1)]^(y[j,2:K])*(1-cp0[j,1:(K-1)])^(1-y[j,2:K]))))^(1-y[j,8])))^(1-y[j,9])
)^((y[j,10]==4)*1)
```

```
    #Modeling pr(y_j = + | y_j-1 ... y_1, D=+)
    cp1[j,1]  =  phi( inprod( y[j,c(1,8,9,11:13)], beta[1:6,1] ) )
    cp1[j,2]  =  phi( inprod( y[j,c(1,8,9,11:13)], beta[1:6,2] ) )
    cp1[j,3]  =  phi( inprod( y[j,c(1,3,8,9,11:13)], beta[1:7,3] ) )
    cp1[j,4]  =  phi( inprod( y[j,c(1,3:4,8,9,11:13)], beta[1:8,4] ) )
    cp1[j,5]  =  phi( inprod( y[j,c(1,8,9,11:13)], beta[1:6,5] ) )
    cp1[j,6]  =  phi( inprod( y[j,c(1,6,8,9,11:13)], beta[1:7,6] ) )
    #Conditional probabilities
    jp1x[j,1] = y[j,2]*cp1[j,1] + (1-y[j,2])*(1-cp1[j,1])
    jp1x[j,2] = y[j,3]*cp1[j,2] + (1-y[j,3])*(1-cp1[j,2])
    jp1x[j,3] = y[j,4]*cp1[j,3] + (1-y[j,4])*(1-cp1[j,3])
    jp1x[j,4] = y[j,5]*cp1[j,4] + (1-y[j,5])*(1-cp1[j,4])
    jp1x[j,5] = y[j,6]*cp1[j,5] + (1-y[j,6])*(1-cp1[j,5])
    jp1x[j,6] = y[j,7]*cp1[j,6] + (1-y[j,7])*(1-cp1[j,6])

    #Modeling pr(y_j = + | y_j-1 ... y_1, D=-)
    cp0[j,1]  =  phi( inprod( y[j,c(1,8,9,11:13)], alpha[1:6,1] ) )
    cp0[j,2]  =  phi( inprod( y[j,c(1:2,8,9,11:13)], alpha[1:7,2] ) )
    cp0[j,3]  =  phi( inprod( y[j,c(1:3,8,9,11:13)], alpha[1:8,3] ) )
    cp0[j,4]  =  phi( inprod( y[j,c(1:4,8,9,11:13)], alpha[1:9,4] ) )
    cp0[j,5]  =  phi( inprod( y[j,c(1)], alpha[1:1,5] ) )
    cp0[j,6]  =  phi( inprod( y[j,c(1)], alpha[1:1,6] ) )

    #Conditional probabilities
    jp0x[j,1] = y[j,2]*cp0[j,1] + (1-y[j,2])*(1 - cp0[j,1])
    jp0x[j,2] = y[j,3]*cp0[j,2] + (1-y[j,3])*(1 - cp0[j,2])
    jp0x[j,3] = y[j,4]*cp0[j,3] + (1-y[j,4])*(1 - cp0[j,3])
    jp0x[j,4] = y[j,5]*cp0[j,4] + (1-y[j,5])*(1 - cp0[j,4])
    jp0x[j,5] = y[j,6]*cp0[j,5] + (1-y[j,6])*(1 - cp0[j,5])
    jp0x[j,6] = y[j,7]*cp0[j,6] + (1-y[j,7])*(1 - cp0[j,6])

    jp1[j,1] = prod( jp1x[j,c(1:1)] )
    jp1[j,2] = prod( jp1x[j,c(2:2)] )
    jp1[j,3] = prod( jp1x[j,c(2:3)] )
    jp1[j,4] = prod( jp1x[j,c(2:4)] )
    jp1[j,5] = prod( jp1x[j,c(5:5)] )
    jp1[j,6] = prod( jp1x[j,c(5:6)] )

    jp0[j,1] = prod( jp0x[j,c(1:1)] )
    jp0[j,2] = prod( jp0x[j,c(1:2)] )
    jp0[j,3] = prod( jp0x[j,c(1:3)] )
    jp0[j,4] = prod( jp0x[j,c(1:4)] )
    jp0[j,5] = prod( jp0x[j,c(5:5)] )
    jp0[j,6] = prod( jp0x[j,c(6:6)] )

    #Cond. prob of TB given HIV, sex, age
    pij[j] <- phi( a_prev[1] + a_prev[2]*y[j,8]  + a_prev[3]*y[j,9] + a_prev[4]*y[j,11] +
                  a_prev[5]*y[j,12] + a_prev[6]*y[j,13] )
    #Cond. prob. of HIV+ given sex, age
    gammaj[j] = phi( b.x[1] + b.x[2]*y[j,9] + b.x[3]*y[j,11] + b.x[4]*y[j,12] + b.x[5]*y[j,13] )
    #Cond. prob of male given age
    psij[j] = phi( z.x[1] + z.x[2]*y[j,11] + z.x[3]*y[j,12] + z.x[4]*y[j,13])
  }
```

```
  pi[1] = phi( sum( a_prev[c(1,2,3)]) )      #TB+, HIV+ male aged 15-29
  pi[2] = phi( sum( a_prev[c(1,3)]) )        #TB+, HIV- male aged 15-29
  pi[3] = phi( sum( a_prev[c(1,2)]  ) )       #TB+, HIV+ female aged 15-29
  pi[4] = phi( sum( a_prev[c(1)]  ) )         #TB+, HIV- female aged 15-29

  pi[5] = phi( sum( a_prev[c(1,2,3,4)] ) )    #TB+, HIV+ male aged 30-49
  pi[6] = phi( sum( a_prev[c(1,3,4)] ) )      #TB+, HIV- male aged 30-49
  pi[7] = phi( sum( a_prev[c(1,2,4)] ) )      #TB+, HIV+ female aged 30-49
  pi[8] = phi( sum( a_prev[c(1,4)] ) )        #TB+, HIV- female aged 30-49

  pi[9] = phi( sum( a_prev[c(1,2,3,5)]  ) )    #TB+, HIV+ male aged 50-69
  pi[10] = phi( sum( a_prev[c(1,3,5)]  ) )     #TB+, HIV- male aged 50-69
  pi[11] = phi( sum( a_prev[c(1,2,5)]  ) )     #TB+, HIV+ female aged 50-69
  pi[12] = phi( sum( a_prev[c(1,5)]  ) )       #TB+, HIV- female aged 50-69

  pi[13] = phi( sum( a_prev[c(1,2,3,6)]  ) )   #TB+, HIV+ male aged 70+
  pi[14] = phi( sum( a_prev[c(1,3,6)]  ) )     #TB+, HIV- male aged 70+
  pi[15] = phi( sum( a_prev[c(1,2,6)]  ) )     #TB+, HIV+ female aged 70+
  pi[16] = phi( sum( a_prev[c(1,6)]  ) )       #TB+, HIV- female aged 70+

  gamma[1] = phi( sum( b.x[c(1,2)]  ) )    #HIV+ male aged 15-29
  gamma[2] = phi( sum( b.x[c(1)]  ) )      #HIV+ female aged 15-29
  gamma[3] = phi( sum( b.x[c(1,2,3)]  ) )  #HIV+ male aged 30-49
  gamma[4] = phi( sum( b.x[c(1,3)]  ) )    #HIV+ female aged 30-49
  gamma[5] = phi( sum( b.x[c(1,2,4)]  ) )  #HIV+ male aged 50-69
  gamma[6] = phi( sum( b.x[c(1,4)]  ) )    #HIV+ female aged 50-69
  gamma[7] = phi( sum( b.x[c(1,2,5)]  ) )  #HIV+ male aged 70+
  gamma[8] = phi( sum( b.x[c(1,5)]  ) )    #HIV+ female aged 70+

  psi[1] = phi( z.x[1] )               #Male aged 15-29
  psi[2] = phi( sum( z.x[c(1,2)] ) )    #Male aged 30-49
  psi[3] = phi( sum( z.x[c(1,3)] ) )    #Male aged 50-69
  psi[4] = phi( sum( z.x[c(1,4)] ) )    #Male aged 70+

  # Total/Overall TB prevalence
  pit[1] =(pi[1]*gamma[1]*psi[1]*pa[1] + pi[2]*(1-gamma[1])*psi[1]*pa[1] + pi[3]*gamma[2]*(1-psi[1])*pa[1] +
pi[4]*(1-gamma[2])*(1-psi[1])*pa[1] +
          pi[5]*gamma[3]*psi[2]*pa[2] + pi[6]*(1-gamma[3])*psi[2]*pa[2] + pi[7]*gamma[4]*(1-psi[2])*pa[2] +
pi[8]*(1-gamma[4])*(1-psi[2])*pa[2] +
          pi[9]*gamma[5]*psi[3]*pa[3] + pi[10]*(1-gamma[5])*psi[3]*pa[3] + pi[11]*gamma[6]*(1-psi[3])*pa[3] +
pi[12]*(1-gamma[6])*(1-psi[3])*pa[3] +
          pi[13]*gamma[7]*psi[4]*pa[4] + pi[14]*(1-gamma[7])*psi[4]*pa[4] + pi[15]*gamma[8]*(1-psi[4])*pa[4] +
pi[16]*(1-gamma[8])*(1-psi[4])*pa[4]) /
   ( gamma[1]*psi[1]*pa[1] + (1-gamma[1])*psi[1]*pa[1] + gamma[2]*(1-psi[1])*pa[1] + (1-gamma[2])*(1-
psi[1])*pa[1] +
      gamma[3]*psi[2]*pa[2] + (1-gamma[3])*psi[2]*pa[2] + gamma[4]*(1-psi[2])*pa[2] + (1-gamma[4])*(1-
psi[2])*pa[2] +
      gamma[5]*psi[3]*pa[3] + (1-gamma[5])*psi[3]*pa[3] + gamma[6]*(1-psi[3])*pa[3] + (1-gamma[6])*(1-
psi[3])*pa[3] +
      gamma[7]*psi[4]*pa[4] + (1-gamma[7])*psi[4]*pa[4] + gamma[8]*(1-psi[4])*pa[4] + (1-gamma[8])*(1-
psi[4])*pa[4])
  #TB in HIV+
```

```
  pit[2] = (pi[1]*gamma[1]*psi[1]*pa[1] + pi[3]*gamma[2]*(1-psi[1])*pa[1] + pi[5]*gamma[3]*psi[2]*pa[2] +
pi[7]*gamma[4]*(1-psi[2])*pa[2] +
           pi[9]*gamma[5]*psi[3]*pa[3] + pi[11]*gamma[6]*(1-psi[3])*pa[3] + pi[13]*gamma[7]*psi[4]*pa[4] +
pi[15]*gamma[8]*(1-psi[4])*pa[4])/
    (gamma[1]*psi[1]*pa[1] + gamma[2]*(1-psi[1])*pa[1] + gamma[3]*psi[2]*pa[2] + gamma[4]*(1-psi[2])*pa[2] +
      gamma[5]*psi[3]*pa[3] + gamma[6]*(1-psi[3])*pa[3] + gamma[7]*psi[4]*pa[4] + gamma[8]*(1-psi[4])*pa[4])
  #TB in HIV-
  pit[3] =(pi[2]*(1-gamma[1])*psi[1]*pa[1] + pi[4]*(1-gamma[2])*(1-psi[1])*pa[1] +  pi[6]*(1-
gamma[3])*psi[2]*pa[2] + pi[8]*(1-gamma[4])*(1-psi[2])*pa[2] +
          pi[10]*(1-gamma[5])*psi[3]*pa[3] + pi[12]*(1-gamma[6])*(1-psi[3])*pa[3] + pi[14]*(1-
gamma[7])*psi[4]*pa[4] + pi[16]*(1-gamma[8])*(1-psi[4])*pa[4])/
    ((1-gamma[1])*psi[1]*pa[1] + (1-gamma[2])*(1-psi[1])*pa[1] +  (1-gamma[3])*psi[2]*pa[2] + (1-gamma[4])*(1-
psi[2])*pa[2] +
      (1-gamma[5])*psi[3]*pa[3] + (1-gamma[6])*(1-psi[3])*pa[3] + (1-gamma[7])*psi[4]*pa[4] + (1-gamma[8])*(1-
psi[4])*pa[4])

  #TB in male
  pit[4] =(pi[1]*gamma[1]*psi[1]*pa[1] + pi[2]*(1-gamma[1])*psi[1]*pa[1] + pi[5]*gamma[3]*psi[2]*pa[2] +
pi[6]*(1-gamma[3])*psi[2]*pa[2] +
          pi[9]*gamma[5]*psi[3]*pa[3] + pi[10]*(1-gamma[5])*psi[3]*pa[3] + pi[13]*gamma[7]*psi[4]*pa[4] +
pi[14]*(1-gamma[7])*psi[4]*pa[4] )/
    (gamma[1]*psi[1]*pa[1] + (1-gamma[1])*psi[1]*pa[1] + gamma[3]*psi[2]*pa[2] + (1-gamma[3])*psi[2]*pa[2] +
      gamma[5]*psi[3]*pa[3] + (1-gamma[5])*psi[3]*pa[3] + gamma[7]*psi[4]*pa[4] + (1-gamma[7])*psi[4]*pa[4])
  #TB in female
  pit[5] = (pi[3]*gamma[2]*(1-psi[1])*pa[1] + pi[4]*(1-gamma[2])*(1-psi[1])*pa[1] +  pi[7]*gamma[4]*(1-
psi[2])*pa[2] + pi[8]*(1-gamma[4])*(1-psi[2])*pa[2] +
           pi[11]*gamma[6]*(1-psi[3])*pa[3] + pi[12]*(1-gamma[6])*(1-psi[3])*pa[3] + pi[15]*gamma[8]*(1-
psi[4])*pa[4] + pi[16]*(1-gamma[8])*(1-psi[4])*pa[4])/
    (gamma[2]*(1-psi[1])*pa[1] + (1-gamma[2])*(1-psi[1])*pa[1] +  gamma[4]*(1-psi[2])*pa[2] + (1-gamma[4])*(1-
psi[2])*pa[2] +
      gamma[6]*(1-psi[3])*pa[3] + (1-gamma[6])*(1-psi[3])*pa[3] + gamma[8]*(1-psi[4])*pa[4] + (1-gamma[8])*(1-
psi[4])*pa[4])
  # TB in each age group
  pit[6] = (pi[1]*gamma[1]*psi[1]*pa[1] + pi[2]*(1-gamma[1])*psi[1]*pa[1] + pi[3]*gamma[2]*(1-psi[1])*pa[1] +
pi[4]*(1-gamma[2])*(1-psi[1])*pa[1])/
    (gamma[1]*psi[1]*pa[1] + (1-gamma[1])*psi[1]*pa[1] + gamma[2]*(1-psi[1])*pa[1] + (1-gamma[2])*(1-
psi[1])*pa[1])
  pit[7] = (pi[5]*gamma[3]*psi[2]*pa[2] + pi[6]*(1-gamma[3])*psi[2]*pa[2] + pi[7]*gamma[4]*(1-psi[2])*pa[2] +
pi[8]*(1-gamma[4])*(1-psi[2])*pa[2])/
    (gamma[3]*psi[2]*pa[2] + (1-gamma[3])*psi[2]*pa[2] + gamma[4]*(1-psi[2])*pa[2] + (1-gamma[4])*(1-
psi[2])*pa[2])
  pit[8] = (pi[9]*gamma[5]*psi[3]*pa[3] + pi[10]*(1-gamma[5])*psi[3]*pa[3] + pi[11]*gamma[6]*(1-psi[3])*pa[3] +
pi[12]*(1-gamma[6])*(1-psi[3])*pa[3])/
    (gamma[5]*psi[3]*pa[3] + (1-gamma[5])*psi[3]*pa[3] + gamma[6]*(1-psi[3])*pa[3] + (1-gamma[6])*(1-
psi[3])*pa[3])
  pit[9] = (pi[13]*gamma[7]*psi[4]*pa[4] + pi[14]*(1-gamma[7])*psi[4]*pa[4] + pi[15]*gamma[8]*(1-psi[4])*pa[4] +
pi[16]*(1-gamma[8])*(1-psi[4])*pa[4])/
    (gamma[7]*psi[4]*pa[4] + (1-gamma[7])*psi[4]*pa[4] + gamma[8]*(1-psi[4])*pa[4] + (1-gamma[8])*(1-
psi[4])*pa[4])

  se[1,1] = sum( jp1[c(1:1), 1] )
  se[2,1] = sum( jp1[c(1:1), 2] )
  se[3,1] = sum( jp1[c(1,3), 3] )
```

```
  se[4,1] = sum( jp1[c(1,3,5,7), 4] )
  se[5,1] = sum( jp1[c(1:1), 5] )
  se[6,1] = sum( jp1[c(1,17), 6] )

  sp[1,1] = 1 - sum( jp0[c(1:1), 1] )
  sp[2,1] = 1 - sum( jp0[c(1:2), 2] )
  sp[3,1] = 1 - sum( jp0[c(1:4), 3] )
  sp[4,1] = 1 - sum( jp0[c(1:8), 4] )
  sp[5,1] = 1 - sum( jp0[c(1:1), 5] )
  sp[6,1] = 1 - sum( jp0[c(1:1), 6] )
  for(g in c(1:15)){
    se[1,g+1] = sum( jp1[c(1:1)+g*2^(K-1), 1] )
    se[2,g+1] = sum( jp1[c(1:1)+g*2^(K-1), 2] )
    se[3,g+1] = sum( jp1[c(1,3)+g*2^(K-1), 3] )
    se[4,g+1] = sum( jp1[c(1,3,5,7)+g*2^(K-1), 4] )
    se[5,g+1] = sum( jp1[c(1)+g*2^(K-1), 5] )
    se[6,g+1] = sum( jp1[c(1,17)+g*2^(K-1), 6] )

    sp[1,g+1] = 1 - sum( jp0[c(1:1)+g*2^(K-1), 1] )
    sp[2,g+1] = 1 - sum( jp0[c(1:2)+g*2^(K-1), 2] )
    sp[3,g+1] = 1 - sum( jp0[c(1:4)+g*2^(K-1), 3] )
    sp[4,g+1] = 1 - sum( jp0[c(1:8)+g*2^(K-1), 4] )
    sp[5,g+1] = 1 - sum( jp0[c(1:1)+g*2^(K-1), 5] )
    sp[6,g+1] = 1 - sum( jp0[c(1:1)+g*2^(K-1), 6] )
  }

  for(g in 1:6){
    # Total/Overall sensitivity
    set[g,1] =(se[g,1]*gamma[1]*psi[1]*pa[1] + se[g,2]*(1-gamma[1])*psi[1]*pa[1] + se[g,3]*gamma[2]*(1-
psi[1])*pa[1] + se[g,4]*(1-gamma[2])*(1-psi[1])*pa[1] +
                se[g,5]*gamma[3]*psi[2]*pa[2] + se[g,6]*(1-gamma[3])*psi[2]*pa[2] + se[g,7]*gamma[4]*(1-
psi[2])*pa[2] + se[g,8]*(1-gamma[4])*(1-psi[2])*pa[2] +
                se[g,9]*gamma[5]*psi[3]*pa[3] + se[g,10]*(1-gamma[5])*psi[3]*pa[3] + se[g,11]*gamma[6]*(1-
psi[3])*pa[3] + se[g,12]*(1-gamma[6])*(1-psi[3])*pa[3] +
                se[g,13]*gamma[7]*psi[4]*pa[4] + se[g,14]*(1-gamma[7])*psi[4]*pa[4] + se[g,15]*gamma[8]*(1-
psi[4])*pa[4] + se[g,16]*(1-gamma[8])*(1-psi[4])*pa[4]) /
      ( gamma[1]*psi[1]*pa[1] + (1-gamma[1])*psi[1]*pa[1] + gamma[2]*(1-psi[1])*pa[1] + (1-gamma[2])*(1-
psi[1])*pa[1] +
          gamma[3]*psi[2]*pa[2] + (1-gamma[3])*psi[2]*pa[2] + gamma[4]*(1-psi[2])*pa[2] + (1-gamma[4])*(1-
psi[2])*pa[2] +
          gamma[5]*psi[3]*pa[3] + (1-gamma[5])*psi[3]*pa[3] + gamma[6]*(1-psi[3])*pa[3] + (1-gamma[6])*(1-
psi[3])*pa[3] +
          gamma[7]*psi[4]*pa[4] + (1-gamma[7])*psi[4]*pa[4] + gamma[8]*(1-psi[4])*pa[4] + (1-gamma[8])*(1-
psi[4])*pa[4])

    #sensitivity in HIV+
    set[g,2] = (se[g,1]*gamma[1]*psi[1]*pa[1] + se[g,3]*gamma[2]*(1-psi[1])*pa[1] +
se[g,5]*gamma[3]*psi[2]*pa[2] + se[g,7]*gamma[4]*(1-psi[2])*pa[2] +
                 se[g,9]*gamma[5]*psi[3]*pa[3] + se[g,11]*gamma[6]*(1-psi[3])*pa[3] + se[g,13]*gamma[7]*psi[4]*pa[4]
+ se[g,15]*gamma[8]*(1-psi[4])*pa[4])/
      (gamma[1]*psi[1]*pa[1] + gamma[2]*(1-psi[1])*pa[1] + gamma[3]*psi[2]*pa[2] + gamma[4]*(1-psi[2])*pa[2] +
        gamma[5]*psi[3]*pa[3] + gamma[6]*(1-psi[3])*pa[3] + gamma[7]*psi[4]*pa[4] + gamma[8]*(1-psi[4])*pa[4])
    #sensitivity in HIV-
```

```
    set[g,3] =(se[g,2]*(1-gamma[1])*psi[1]*pa[1] + se[g,4]*(1-gamma[2])*(1-psi[1])*pa[1] +  se[g,6]*(1-gamma[3])*psi[2]*pa[2] + se[g,8]*(1-gamma[4])*(1-psi[2])*pa[2] +
              se[g,10]*(1-gamma[5])*psi[3]*pa[3] + se[g,12]*(1-gamma[6])*(1-psi[3])*pa[3] + se[g,14]*(1-gamma[7])*psi[4]*pa[4] + se[g,16]*(1-gamma[8])*(1-psi[4])*pa[4])/
      ((1-gamma[1])*psi[1]*pa[1] + (1-gamma[2])*(1-psi[1])*pa[1] +  (1-gamma[3])*psi[2]*pa[2] + (1-gamma[4])*(1-psi[2])*pa[2] +
        (1-gamma[5])*psi[3]*pa[3] + (1-gamma[6])*(1-psi[3])*pa[3] + (1-gamma[7])*psi[4]*pa[4] + (1-gamma[8])*(1-psi[4])*pa[4])



    #sensitivity in male
    set[g,4] =(se[g,1]*gamma[1]*psi[1]*pa[1] + se[g,2]*(1-gamma[1])*psi[1]*pa[1] + se[g,5]*gamma[3]*psi[2]*pa[2] + se[g,6]*(1-gamma[3])*psi[2]*pa[2] +
              se[g,9]*gamma[5]*psi[3]*pa[3] + se[g,10]*(1-gamma[5])*psi[3]*pa[3] + se[g,13]*gamma[7]*psi[4]*pa[4] + se[g,14]*(1-gamma[7])*psi[4]*pa[4] )/
      (gamma[1]*psi[1]*pa[1] + (1-gamma[1])*psi[1]*pa[1] + gamma[3]*psi[2]*pa[2] + (1-gamma[3])*psi[2]*pa[2] +
        gamma[5]*psi[3]*pa[3] + (1-gamma[5])*psi[3]*pa[3] + gamma[7]*psi[4]*pa[4] + (1-gamma[7])*psi[4]*pa[4])
    #TB in female
    set[g,5] = (se[g,3]*gamma[2]*(1-psi[1])*pa[1] + se[g,4]*(1-gamma[2])*(1-psi[1])*pa[1] +  se[g,7]*gamma[4]*(1-psi[2])*pa[2] + se[g,8]*(1-gamma[4])*(1-psi[2])*pa[2] +
               se[g,11]*gamma[6]*(1-psi[3])*pa[3] + se[g,12]*(1-gamma[6])*(1-psi[3])*pa[3] + se[g,15]*gamma[8]*(1-psi[4])*pa[4] + se[g,16]*(1-gamma[8])*(1-psi[4])*pa[4])/
      (gamma[2]*(1-psi[1])*pa[1] + (1-gamma[2])*(1-psi[1])*pa[1] +  gamma[4]*(1-psi[2])*pa[2] + (1-gamma[4])*(1-psi[2])*pa[2] +
        gamma[6]*(1-psi[3])*pa[3] + (1-gamma[6])*(1-psi[3])*pa[3] + gamma[8]*(1-psi[4])*pa[4] + (1-gamma[8])*(1-psi[4])*pa[4])
    # sensitivity in each age group
    set[g,6] = (se[g,1]*gamma[1]*psi[1]*pa[1] + se[g,2]*(1-gamma[1])*psi[1]*pa[1] + se[g,3]*gamma[2]*(1-psi[1])*pa[1] + se[g,4]*(1-gamma[2])*(1-psi[1])*pa[1])/
      (gamma[1]*psi[1]*pa[1] + (1-gamma[1])*psi[1]*pa[1] + gamma[2]*(1-psi[1])*pa[1] + (1-gamma[2])*(1-psi[1])*pa[1])
    set[g,7] = (se[g,5]*gamma[3]*psi[2]*pa[2] + se[g,6]*(1-gamma[3])*psi[2]*pa[2] + se[g,7]*gamma[4]*(1-psi[2])*pa[2] + se[g,8]*(1-gamma[4])*(1-psi[2])*pa[2])/
      (gamma[3]*psi[2]*pa[2] + (1-gamma[3])*psi[2]*pa[2] + gamma[4]*(1-psi[2])*pa[2] + (1-gamma[4])*(1-psi[2])*pa[2])
    set[g,8] = (se[g,9]*gamma[5]*psi[3]*pa[3] + se[g,10]*(1-gamma[5])*psi[3]*pa[3] + se[g,11]*gamma[6]*(1-psi[3])*pa[3] + se[g,12]*(1-gamma[6])*(1-psi[3])*pa[3])/
      (gamma[5]*psi[3]*pa[3] + (1-gamma[5])*psi[3]*pa[3] + gamma[6]*(1-psi[3])*pa[3] + (1-gamma[6])*(1-psi[3])*pa[3])
    set[g,9] = (se[g,13]*gamma[7]*psi[4]*pa[4] + se[g,14]*(1-gamma[7])*psi[4]*pa[4] + se[g,15]*gamma[8]*(1-psi[4])*pa[4] + se[g,16]*(1-gamma[8])*(1-psi[4])*pa[4])/
      (gamma[7]*psi[4]*pa[4] + (1-gamma[7])*psi[4]*pa[4] + gamma[8]*(1-psi[4])*pa[4] + (1-gamma[8])*(1-psi[4])*pa[4])


    # Total/Overall specificity
    spt[g,1] =(sp[g,1]*gamma[1]*psi[1]*pa[1] + sp[g,2]*(1-gamma[1])*psi[1]*pa[1] + sp[g,3]*gamma[2]*(1-psi[1])*pa[1] + sp[g,4]*(1-gamma[2])*(1-psi[1])*pa[1] +
              sp[g,5]*gamma[3]*psi[2]*pa[2] + sp[g,6]*(1-gamma[3])*psi[2]*pa[2] + sp[g,7]*gamma[4]*(1-psi[2])*pa[2] + sp[g,8]*(1-gamma[4])*(1-psi[2])*pa[2] +
              sp[g,9]*gamma[5]*psi[3]*pa[3] + sp[g,10]*(1-gamma[5])*psi[3]*pa[3] + sp[g,11]*gamma[6]*(1-psi[3])*pa[3] + sp[g,12]*(1-gamma[6])*(1-psi[3])*pa[3] +
```

```
            sp[g,13]*gamma[7]*psi[4]*pa[4] + sp[g,14]*(1-gamma[7])*psi[4]*pa[4] + sp[g,15]*gamma[8]*(1-
psi[4])*pa[4] + sp[g,16]*(1-gamma[8])*(1-psi[4])*pa[4]) /
   ( gamma[1]*psi[1]*pa[1] + (1-gamma[1])*psi[1]*pa[1] + gamma[2]*(1-psi[1])*pa[1] + (1-gamma[2])*(1-
psi[1])*pa[1] +
     gamma[3]*psi[2]*pa[2] + (1-gamma[3])*psi[2]*pa[2] + gamma[4]*(1-psi[2])*pa[2] + (1-gamma[4])*(1-
psi[2])*pa[2] +
     gamma[5]*psi[3]*pa[3] + (1-gamma[5])*psi[3]*pa[3] + gamma[6]*(1-psi[3])*pa[3] + (1-gamma[6])*(1-
psi[3])*pa[3] +
     gamma[7]*psi[4]*pa[4] + (1-gamma[7])*psi[4]*pa[4] + gamma[8]*(1-psi[4])*pa[4] + (1-gamma[8])*(1-
psi[4])*pa[4])


  #specificity  in HIV+
  spt[g,2] = (sp[g,1]*gamma[1]*psi[1]*pa[1] + sp[g,3]*gamma[2]*(1-psi[1])*pa[1] +
sp[g,5]*gamma[3]*psi[2]*pa[2] + sp[g,7]*gamma[4]*(1-psi[2])*pa[2] +
            sp[g,9]*gamma[5]*psi[3]*pa[3] + sp[g,11]*gamma[6]*(1-psi[3])*pa[3] + sp[g,13]*gamma[7]*psi[4]*pa[4]
+ sp[g,15]*gamma[8]*(1-psi[4])*pa[4])/
   (gamma[1]*psi[1]*pa[1] + gamma[2]*(1-psi[1])*pa[1] + gamma[3]*psi[2]*pa[2] + gamma[4]*(1-psi[2])*pa[2] +
     gamma[5]*psi[3]*pa[3] + gamma[6]*(1-psi[3])*pa[3] + gamma[7]*psi[4]*pa[4] + gamma[8]*(1-psi[4])*pa[4])
  #specificity  in HIV-
  spt[g,3] =(sp[g,2]*(1-gamma[1])*psi[1]*pa[1] + sp[g,4]*(1-gamma[2])*(1-psi[1])*pa[1] +  sp[g,6]*(1-
gamma[3])*psi[2]*pa[2] + sp[g,8]*(1-gamma[4])*(1-psi[2])*pa[2] +
            sp[g,10]*(1-gamma[5])*psi[3]*pa[3] + sp[g,12]*(1-gamma[6])*(1-psi[3])*pa[3] + sp[g,14]*(1-
gamma[7])*psi[4]*pa[4] + sp[g,16]*(1-gamma[8])*(1-psi[4])*pa[4])/
   ((1-gamma[1])*psi[1]*pa[1] + (1-gamma[2])*(1-psi[1])*pa[1] +  (1-gamma[3])*psi[2]*pa[2] + (1-gamma[4])*(1-
psi[2])*pa[2] +
     (1-gamma[5])*psi[3]*pa[3] + (1-gamma[6])*(1-psi[3])*pa[3] + (1-gamma[7])*psi[4]*pa[4] + (1-gamma[8])*(1-
psi[4])*pa[4])

  #specificity  in male
  spt[g,4] =(sp[g,1]*gamma[1]*psi[1]*pa[1] + sp[g,2]*(1-gamma[1])*psi[1]*pa[1] + sp[g,5]*gamma[3]*psi[2]*pa[2]
+ sp[g,6]*(1-gamma[3])*psi[2]*pa[2] +
            sp[g,9]*gamma[5]*psi[3]*pa[3] + sp[g,10]*(1-gamma[5])*psi[3]*pa[3] + sp[g,13]*gamma[7]*psi[4]*pa[4]
+ sp[g,14]*(1-gamma[7])*psi[4]*pa[4] )/
   (gamma[1]*psi[1]*pa[1] + (1-gamma[1])*psi[1]*pa[1] + gamma[3]*psi[2]*pa[2] + (1-gamma[3])*psi[2]*pa[2] +
     gamma[5]*psi[3]*pa[3] + (1-gamma[5])*psi[3]*pa[3] + gamma[7]*psi[4]*pa[4] + (1-gamma[7])*psi[4]*pa[4])
  #specificity in female
  spt[g,5] = (sp[g,3]*gamma[2]*(1-psi[1])*pa[1] + sp[g,4]*(1-gamma[2])*(1-psi[1])*pa[1] +  sp[g,7]*gamma[4]*(1-
psi[2])*pa[2] + sp[g,8]*(1-gamma[4])*(1-psi[2])*pa[2] +
            sp[g,11]*gamma[6]*(1-psi[3])*pa[3] + sp[g,12]*(1-gamma[6])*(1-psi[3])*pa[3] + sp[g,15]*gamma[8]*(1-
psi[4])*pa[4] + sp[g,16]*(1-gamma[8])*(1-psi[4])*pa[4])/
   (gamma[2]*(1-psi[1])*pa[1] + (1-gamma[2])*(1-psi[1])*pa[1] +  gamma[4]*(1-psi[2])*pa[2] + (1-gamma[4])*(1-
psi[2])*pa[2] +
     gamma[6]*(1-psi[3])*pa[3] + (1-gamma[6])*(1-psi[3])*pa[3] + gamma[8]*(1-psi[4])*pa[4] + (1-gamma[8])*(1-
psi[4])*pa[4])
  # specificity  in each age group
  spt[g,6] = (sp[g,1]*gamma[1]*psi[1]*pa[1] + sp[g,2]*(1-gamma[1])*psi[1]*pa[1] + sp[g,3]*gamma[2]*(1-
psi[1])*pa[1] + sp[g,4]*(1-gamma[2])*(1-psi[1])*pa[1])/
   (gamma[1]*psi[1]*pa[1] + (1-gamma[1])*psi[1]*pa[1] + gamma[2]*(1-psi[1])*pa[1] + (1-gamma[2])*(1-
psi[1])*pa[1])
  spt[g,7] = (sp[g,5]*gamma[3]*psi[2]*pa[2] + sp[g,6]*(1-gamma[3])*psi[2]*pa[2] + sp[g,7]*gamma[4]*(1-
psi[2])*pa[2] + sp[g,8]*(1-gamma[4])*(1-psi[2])*pa[2])/
```

```
      (gamma[3]*psi[2]*pa[2] + (1-gamma[3])*psi[2]*pa[2] + gamma[4]*(1-psi[2])*pa[2] + (1-gamma[4])*(1-
psi[2])*pa[2])
    spt[g,8] = (sp[g,9]*gamma[5]*psi[3]*pa[3] + sp[g,10]*(1-gamma[5])*psi[3]*pa[3] + sp[g,11]*gamma[6]*(1-
psi[3])*pa[3] + sp[g,12]*(1-gamma[6])*(1-psi[3])*pa[3])/
      (gamma[5]*psi[3]*pa[3] + (1-gamma[5])*psi[3]*pa[3] + gamma[6]*(1-psi[3])*pa[3] + (1-gamma[6])*(1-
psi[3])*pa[3])
    spt[g,9] = (sp[g,13]*gamma[7]*psi[4]*pa[4] + sp[g,14]*(1-gamma[7])*psi[4]*pa[4] + sp[g,15]*gamma[8]*(1-
psi[4])*pa[4] + sp[g,16]*(1-gamma[8])*(1-psi[4])*pa[4])/
      (gamma[7]*psi[4]*pa[4] + (1-gamma[7])*psi[4]*pa[4] + gamma[8]*(1-psi[4])*pa[4] + (1-gamma[8])*(1-
psi[4])*pa[4])
  }

  ## priors ------------------------------------------------------------------------------------------
  for(g in 1:(K-1)){
    for(r in 1:g1[g]){
      beta[r,g] ~ dnorm(mu1[r,g], s1[r,g])
    }
  }
  for(g in 1:(K-1)){
    for(r in 1:g0[g]){
      alpha[r,g] ~ dnorm(mu0[r,g], s0[r,g])
    }
  }
  # Prior for prevalence
  a_prev[1] ~ dnorm( mu.prev[1], s.prev[1] )
  a_prev[2] ~ dnorm( mu.prev[2], s.prev[2] )
  a_prev[3] ~ dnorm( mu.prev[3], s.prev[3] )
  a_prev[4] ~ dnorm( mu.prev[4], s.prev[4] )
  a_prev[5] ~ dnorm( mu.prev[5], s.prev[5] )
  a_prev[6] ~ dnorm( mu.prev[6], s.prev[6] )

  b.x[1] ~ dnorm( mu.x[1], s.x[1] )
  b.x[2] ~ dnorm( mu.x[2], s.x[2] )
  b.x[3] ~ dnorm( mu.x[3], s.x[3] )
  b.x[4] ~ dnorm( mu.x[4], s.x[4] )
  b.x[5] ~ dnorm( mu.x[5], s.x[5] )

  z.x[1] ~ dnorm( mu.z[1], s.z[1] )
  z.x[2] ~ dnorm( mu.z[2], s.z[2] )
  z.x[3] ~ dnorm( mu.z[3], s.z[3] )
  z.x[4] ~ dnorm( mu.z[4], s.z[4] )

  pa[1:4] ~ ddirch( mu.a[1:4] )
}
#==========================================================================

#Choice of priors (Determined outside jags and issued as part of the data)
g1 = c(6,6,7,8,6,7)  # Number of parameters in models for non-PTB cases
g0 = c(6,7,8,9,1,1)  # Number of parameters in models for non-PTB cases

mu1 = matrix(NA, nrow = max(g1), ncol = 6)  #Mean among PTB cases
s1 = matrix(NA, nrow = max(g1), ncol = 6)     # Precision for mean among the PTB cases

```

```
for(c in 1:ncol(mu1)){
  for(r in 1:g1[c]){
    mu1[r,c] = 0
  }
}
mu1[1,6] = qnorm(0.8)   # Informative prior of the parameter corresponding to Pr(culture =+ | Xpert ultra = -, D+)

for(c in 1:ncol(s1)){
  for(r in 1:g1[c]){
    s1[r,c] = 1
  }
}
s1[1,1] = 10
s1[1,6] = 10  #precision for the informative prior of the parameter corresponding to Pr(cult =+ | Xpert ultra = -, D+)

mu0 = matrix(NA, nrow = max(g0), ncol = 6)  #Mean among non PTB cases
s0 = matrix(NA, nrow = max(g0), ncol = 6)  # Precision for mean among the non PTB cases

for(c in 1:ncol(mu0)){
  for(r in 1:g0[c]){
    mu0[r,c] = 0
  }
}
mu0[1,5] <- -qnorm(0.97) # prior for Pr(Xpert Ultra =+ | D = -)
mu0[1,6] <- -qnorm(0.999) # prior for Pr(culture =+ | D = -)

for(c in 1:ncol(s0)){
  for(r in 1:g0[c]){
    s0[r,c] = .1
  }
}
s0[1,5:6] <- 100 # precision for prior for Pr(Xpert Ultra =+ | D = -) and prior for Pr(culture =+ | D = -) resp.

mu.prev = numeric(6)
s.prev = numeric(6)
mu.prev = c(-5,0,0,0,0,0)
s.prev = c(10,100,100,100,100,100)

#Prior for covariates in the models
mu.x = numeric(5)
s.x = numeric(5)
mu.x = c(0,0,0,0,0)
s.x = c(1,1,1,1,1)
mu.z = c(0,0,0,0)
s.z = c(1,1,1,1)

mu.a = c(1,1,1,1)  # Dirichlet prior for age groups
```

Analysis that replaces any chest X-ray abnormality with chest X-ray abnormality suggestive of active TB

Tables S10 – S14 present the results obtained following the alternative analysis that replaces any chest X-ray abnormality with chest X-ray abnormality suggestive of active TB. Table S15 presents a side-by-side comparison of the overall results from the two alternative analyses.

Table S10: Posterior median and 95% credible intervals (95% CrI) of the prevalence and diagnostic test sensitivity and specificity of Vukuzazi dataset

		Model 0	Model 1	Model 2	Model 3	Model 4
Test	Parameter	Median (95% CrI)	Median (95% CrI)	Median (95% CrI)	Median (95% CrI)	Median (95% CrI)
	Prevalence	15.0 (13.9, 16.2)	16.6 (15.1, 18.5)	1.1 (0.8, 1.5)	1.1 (0.8, 1.5)	1.1 (0.8, 1.6)
Any TB	Sensitivity	22.1 (19.1, 25.1)	21.8 (18.9, 24.8)	27.4 (17.7, 39.5)	27.1 (17.1, 39.3)	27.7 (17.2, 40.1)
symptom	Specificity	83.7 (82.6, 84.8)	83.8 (82.6, 84.9)	83.0 (81.9, 84.0)	83.0 (81.9, 84.0)	83.0 (81.9, 84.0)
Radiologist	Sensitivity	23.1 (20.1, 26.2)	21.2 (18.2, 24.5)	44.1 (32.0, 57.0)	43.6 (31.5, 56.4)	43.7 (31.5, 56.6)
conclusion [Ŧ]	Specificity	99.9 (99.7, 100)	99.9 (99.7, 100)	96.9 (96.4, 97.4)	96.9 (96.4, 97.4)	96.9 (96.4, 97.4)
CAD4TBv5≥53	Sensitivity	95.0 (92.3, 97.1)	88.9 (82.7, 93.7)	80.6 (69.1, 89.3)	80.0 (67.9, 89.0)	80.3 (68.5, 89.1)
	Specificity	92.9 (92.1, 93.8)	93.3 (92.3, 94.5)	80.2 (79.0, 81.3)	80.2 (79.0, 81.3)	80.2 (79.0, 81.3)
CAD4TBv6≥53	Sensitivity	95.1 (92.4, 97.2)	90.5 (84.6, 94.8)	81.4 (70.1, 89.9)	81.3 (69.8, 89.9)	80.9 (69.1, 89.6)
	Specificity	96.0 (95.2, 96.7)	96.6 (95.7, 97.7)	82.7 (81.6, 83.8)	82.7 (81.7, 83.7)	82.8 (81.6, 83.8)
Xpert Ultra[†]	Sensitivity	13.7 (11.5, 16.0)	6.2 (4.7, 8.0)	62.4 (48.9, 75.3)	60.0 (45.6, 73.7)	62.3 (47.5, 76.1)
	Specificity	99.9 (99.8, 100)	99.4 (99.2, 99.6)	99.4 (99.1, 99.6)	99.4 (99.1, 99.6)	99.4 (99.1, 99.6)
Culture	Sensitivity	13.3 (11.2, 15.6)	6.6 (5.0, 8.6)	74.1 (60.2, 86.6)	71.4 (56.4, 85.4)	71.6 (55.8, 85.6)
	Specificity	99.7 (99.5, 99.8)	99.8 (99.7, 99.9)	99.8 (99.7, 99.9)	99.8 (99.7, 99.9)	99.8 (99.7, 99.9)
	Deviance	4823.3	4640.3	4614.7	4610.4	4609.7
	RMSE	126.2	50.8	31.0	30.1	29.8

Model 0 – Based on the assumption of conditional independence
Model 1 – Accounts for conditional dependence between radiologist conclusion, CAD4TBv5≥53 and CAD4TBv6≥53 and between Xpert Ultra and culture among the PTB cases and allows conditional independence between all the diagnostic tests among non-PTB cases
Model 2 – Accounts for conditional dependence between radiologist conclusion, CAD4TBv5≥53 and CAD4TBv6≥53 and between Xpert Ultra and culture among the PTB cases and conditional dependence between any TB symptom, radiologist conclusion, CAD4TBv5≥53 and CAD4TBv6≥53 among non-PTB cases
Model 3 – Accounts for conditional dependence between all the diagnostic tests except any TB symptom among the PTB cases and conditional dependence between any TB symptom, radiologist conclusion, CAD4TBv5≥53 and CAD4TBv6≥53 among non-PTB cases
Model 4 – Accounts for conditional dependence between all the diagnostic tests among the PTB cases and conditional dependence between any TB symptom, radiologist conclusion, CAD4TBv5≥53 and CAD4TBv6≥53 among non-PTB cases
RMSE – Root mean square error. This is calculated as the square root of the sum of squared differences between the observed frequencies and the predicted frequencies. It shows how good the model is in explaining the variability in the data (smaller is better)
CrI – Credible Intervals
Ŧ - Chest X-ray abnormality suggestive of active PTB, † - Excluding trace
Note: The estimates presented in the table are percentages

Table S11: Posterior median and 95% credible intervals (95% CrI) of PTB prevalence and diagnostic test sensitivity and specificity for Vukuzazi dataset adjusted for HIV status, sex and age, by HIV status adjusted for age and sex and by sex adjusted for HIV status and age

		Overall	HIV+	HIV-	Male	Female
	N	4960 (100%)	1496 (30.2%)	3464 (69.8%)	1813 (36.6%)	3147 (63.4%)
Test	Parameter	Median (95% CrI)	Median (95% CrI)	Median (95% CrI)	Median (95% CrI)	Median (95% CrI)
	Prevalence	0.9 (0.6, 1.3)	1.2 (0.8, 1.9)	0.8 (0.5, 1.1)	1.1 (0.7, 1.7)	0.8 (0.5, 1.1)
Any TB	Sensitivity	25.8 (15.9, 38.0)	26.3 (13.5, 43.4)	25.4 (14.8, 39.0)	23.8 (11.5, 39.9)	26.7 (15.7, 40.5)
symptom	Specificity	83.0 (81.9, 84.0)	83.5 (81.5, 85.3)	82.7 (81.4, 83.9)	83.3 (81.5, 84.9)	82.8 (81.4, 84.1)
Radiologist	Sensitivity	44.1 (32.0, 56.8)	44.2 (27.9, 62.1)	43.9 (30.4, 57.9)	50.0 (33.5, 66.6)	40.6 (27.2, 55.2)
conclusion [Ŧ]	Specificity	96.9 (96.3, 97.4)	95.3 (94.1, 96.3)	97.6 (97.0, 98.1)	95.3 (94.3, 96.3)	97.8 (97.2, 98.3)
CAD4TBv5≥53	Sensitivity	81.5 (70.2, 90.2)	84.9 (69.9, 94.5)	80.3 (67.5, 89.8)	85.8 (72.0, 94.5)	79.4 (65.9, 89.6)
	Specificity	80.1 (78.9, 81.2)	78.7 (76.6, 80.8)	80.6 (79.3, 82.0)	72.8 (70.7, 74.9)	84.2 (82.9, 85.4)
CAD4TBv6≥53	Sensitivity	82.5 (71.3, 90.8)	84.3 (69.2, 93.8)	82.1 (69.3, 91.0)	84.4 (69.8, 93.7)	81.8 (69.0, 90.7)
	Specificity	82.6 (81.6, 83.7)	79.2 (77.1, 81.3)	84.1 (82.9, 85.3)	76.2 (74.1, 78.1)	86.4 (85.1, 87.5)
Xpert Ultra[†]	Sensitivity	64.0 (50.6, 76.5)	61.9 (43.7, 78.9)	65.0 (50.1, 78.5)	72.2 (55.8, 86.2)	59.4 (43.8, 74.0)
	Specificity	99.4 (99.1, 99.5)	99.4 (99.1, 99.5)	99.4 (99.1, 99.5)	99.4 (99.1, 99.5)	99.4 (99.1, 99.5)
Culture	Sensitivity	75.2 (61.6, 88.0)	73.4 (53.4, 89.9)	76.3 (61.5, 89.1)	76.7 (59.3, 91.0)	74.7 (59.2, 88.2)
	Specificity	99.8 (99.7, 99.9)	99.8 (99.7, 99.9)	99.8 (99.7, 99.9)	99.8 (99.7, 99.9)	99.8 (99.7, 99.9)

CrI – Credible Intervals

Ŧ - Chest X-ray abnormality suggestive of active PTB

† - Excluding trace

Note: The estimates presented in the table are percentages

Table S12: Age, sex and HIV adjusted posterior median and 95% credible intervals (95% CrI) of PTB prevalence and diagnostic test sensitivity and specificity for Vukuzazi dataset presented by age groups

		15 – 29 years	30 – 49 years	50 – 69 years	≥70 years
	N	1156 (23.3%)	1291 (26.0%)	1723 (34.7%)	790 (15.9%)
Test	Parameter	Median (95% CrI)	Median (95% CrI)	Median (95% CrI)	Median (95% CrI)
	Prevalence	0.8 (0.5, 1.2)	1.0 (0.6, 1.6)	1.0 (0.6, 1.6)	0.7 (0.4, 1.2)
Any TB symptom	Sensitivity	26.5 (15.1, 41.4)	23.1 (9.7, 42.3)	26.9 (12.8, 45.1)	24.9 (9.0, 47.8)
	Specificity	81.5 (79.1, 83.7)	83.7 (81.7, 85.7)	83.5 (81.6, 85.2)	82.8 (80.0, 85.4)
Radiologist conclusion[Ŧ]	Sensitivity	44.3 (30.1, 59.1)	39.7 (21.5, 60.0)	51.5 (33.1, 70.5)	33.5 (15.2, 56.7)
	Specificity	98.4 (97.5, 99.0)	96.3 (95.2, 97.2)	96.1 (95.2, 97.0)	97.4 (96.1, 98.4)
CAD4TBv5≥53	Sensitivity	80.0 (66.4, 90.0)	83.3 (66.2, 94.3)	83.3 (66.5, 93.7)	79.6 (57.8, 93.2)
	Specificity	93.8 (92.3, 95.1)	82.2 (80.0, 84.3)	76.6 (74.5, 78.6)	64.1 (60.7, 67.4)
CAD4TBv6≥53	Sensitivity	81.1 (67.2, 90.7)	82.0 (64.1, 93.2)	85.2 (69.2, 94.6)	82.5 (61.5, 94.5)
	Specificity	95.6 (94.3, 96.7)	83.5 (81.4, 85.5)	78.9 (76.9, 80.8)	70.4 (67.2, 73.4)
Xpert Ultra[†]	Sensitivity	64.5 (48.4, 78.3)	65.2 (44.2, 83.2)	65.7 (45.9, 82.1)	58.9 (35.2, 81.7)
	Specificity	99.4 (99.1, 99.5)	99.4 (99.1, 99.5)	99.4 (99.1, 99.5)	99.4 (99.1, 99.5)
Culture	Sensitivity	77.7 (62.5, 89.6)	77.3 (54.0, 93.4)	70.4 (50.3, 88.3)	81.5 (59.9, 95.0)
	Specificity	99.8 (99.7, 99.9)	99.8 (99.7, 99.9)	99.8 (99.7, 99.9)	99.8 (99.7, 99.9)

CrI – Credible Intervals
Ŧ - Chest X-ray abnormality suggestive of active PTB
† - Excluding trace
Note: The estimates presented in the table are percentages

Table S13: Posterior median and 95% credible intervals (95% CrI) of the age, sex and HIV adjusted PTB prevalence and diagnostic test sensitivity and specificity among male individuals in Vukuzazi dataset

			**15 – 29 years**	**30 – 49 years**	**50 – 69 years**	**≥70 years**
**Group**	**Test**	**Parameter**	**Median (95% CrI)**	**Median (95% CrI)**	**Median (95% CrI)**	**Median (95% CrI)**
**HIV+ Male**		Prevalence	1.4 (0.8, 2.4)	1.5 (0.8, 2.6)	1.9 (1.0, 3.1)	1.6 (0.8, 2.9)
	Any TB	Sensitivity	26.5 (10.2, 49.6)	21.7 (6.8, 46.4)	25.9 (9.5, 48.5)	23.9 (6.4, 53.4)
	symptom	Specificity	82.2 (78.7, 85.5)	84.4 (81.6, 86.9)	84.3 (81.2, 87.1)	83.8 (79.6, 87.4)
	Radiologist	Sensitivity	49.8 (27.6, 72.9)	46.4 (22.3, 71.5)	59.8 (36.4, 80.7)	42.0 (16.2, 72.1)
	conclusion Ŧ	Specificity	95.9 (93.4, 97.6)	92.6 (90.0, 94.6)	90.2 (86.8, 92.9)	91.3 (86.5, 94.8)
	CAD4TBv5≥53	Sensitivity	87.6 (69.3, 96.9)	89.7 (70.6, 98.0)	90.9 (73.9, 98.2)	89.6 (64.9, 98.3)
		Specificity	86.8 (83.3, 89.7)	67.8 (63.9, 71.5)	55.1 (50.4, 59.7)	37.1 (31.4, 43.1)
	CAD4TBv6≥53	Sensitivity	85.7 (65.6, 95.8)	85.8 (63.7, 96.5)	89.2 (71.3, 97.6)	87.7 (62.8, 97.8)
		Specificity	88.9 (85.7, 91.8)	68.6 (64.7, 72.3)	55.6 (50.8, 60.2)	39.9 (34.1, 46.0)
	Xpert Ultra†	Sensitivity	67.9 (43.8, 86.6)	72.2 (46.1, 90.7)	72.5 (48.9, 90.0)	66.3 (36.2, 89.8)
		Specificity	99.4 (99.1, 99.5)	99.4 (99.1, 99.5)	99.4 (99.1, 99.5)	99.4 (99.1, 99.5)
	Culture	Sensitivity	76.7 (51.1, 93.5)	77.5 (48.3, 95.5)	70.0 (44.1, 91.2)	81.0 (50.7, 96.5)
		Specificity	99.8 (99.7, 99.9)	99.8 (99.7, 99.9)	99.8 (99.7, 99.9)	99.8 (99.7, 99.9)
**HIV- Male**		Prevalence	0.9 (0.5, 1.4)	0.9 (0.5, 1.6)	1.2 (0.7, 1.9)	1.0 (0.5, 1.7)
	Any TB	Sensitivity	24.5 (10.7, 43.8)	19.7 (6.4, 42.9)	23.8 (9.2, 45.0)	22.3 (6.3, 47.9)
	symptom	Specificity	81.8 (79.2, 84.3)	84.1 (81.2, 86.6)	83.9 (81.5, 86.2)	83.4 (80.1, 86.4)
	Radiologist	Sensitivity	48.6 (30.1, 67.6)	44.8 (22.6, 69.2)	58.4 (36.1, 78.6)	40.8 (17.6, 67.5)
	conclusionŦ	Specificity	98.0 (96.9, 98.8)	96.1 (94.4, 97.4)	94.7 (92.9, 96.1)	95.3 (93.0, 97.1)
	CAD4TBv5≥53	Sensitivity	83.2 (66.2, 93.9)	85.9 (65.6, 96.3)	87.4 (69.4, 96.6)	85.5 (62.4, 96.6)
		Specificity	91.3 (89.3, 93.2)	75.7 (72.2, 79.2)	64.2 (60.7, 67.7)	45.6 (41.1, 50.0)
	CAD4TBv6≥53	Sensitivity	82.7 (65.0, 93.5)	83.0 (61.3, 95.1)	87.0 (68.8, 96.4)	85.2 (62.6, 96.5)
		Specificity	94.3 (92.5, 95.8)	79.6 (76.2, 82.7)	68.6 (65.1, 71.9)	53.1 (48.4, 57.6)
	Xpert Ultra†	Sensitivity	71.4 (51.6, 86.9)	75.4 (51.1, 92.1)	75.9 (54.7, 90.8)	69.6 (42.8, 90.4)
		Specificity	99.4 (99.1, 99.5)	99.4 (99.1, 99.5)	99.4 (99.1, 99.5)	99.4 (99.1, 99.5)
	Culture	Sensitivity	79.0 (59.2, 93.0)	79.9 (54.3, 95.6)	72.6 (49.4, 91.4)	82.9 (57.9, 96.3)
		Specificity	99.8 (99.7, 99.9)	99.8 (99.7, 99.9)	99.8 (99.7, 99.9)	99.8 (99.7, 99.9)

Ŧ - Chest X-ray abnormality suggestive of active PTB, † - Excluding trace

Table S14: Posterior median and 95% credible intervals (95% CrI) of the age, sex and HIV adjusted PTB prevalence and diagnostic test sensitivity and specificity among female individuals in Vukuzazi dataset

			**15 – 29 years**	**30 – 49 years**	**50 – 69 years**	**≥70 years**
**Group**	**Test**	**Parameter**	**Median (95% CrI)**	**Median (95% CrI)**	**Median (95% CrI)**	**Median (95% CrI)**
**HIV+ Female**		Prevalence	0.9 (0.5, 1.5)	1.0 (0.5, 1.7)	1.2 (0.6, 2.0)	1.0 (0.5, 1.8)
	Any TB	Sensitivity	29.8 (14.1, 49.7)	24.7 (9.4, 47.5)	29.2 (11.9, 51.9)	26.9 (8.8, 54.7)
	symptom	Specificity	81.4 (77.9, 84.6)	83.6 (81.2, 86.0)	83.5 (80.9, 85.9)	83.0 (79.1, 86.4)
	Radiologist	Sensitivity	39.7 (21.5, 60.8)	36.3 (16.7, 60.3)	49.6 (27.9, 72.6)	32.2 (12.0, 60.5)
	conclusion[Ŧ]	Specificity	98.5 (97.3, 99.2)	96.9 (95.7, 97.9)	95.7 (94.1, 97.0)	96.3 (93.9, 97.9)
	CAD4TBv5≥53	Sensitivity	80.6 (61.0, 93.0)	83.4 (62.1, 95.6)	85.1 (64.6, 95.8)	83.0 (57.0, 96.1)
		Specificity	96.1 (94.7, 97.3)	86.9 (84.7, 88.9)	78.6 (75.6, 81.5)	63.0 (57.8, 67.9)
	CAD4TBv6≥53	Sensitivity	82.1 (63.8, 93.2)	82.5 (60.9, 94.6)	86.5 (67.5, 96.4)	84.8 (60.6, 96.4)
		Specificity	96.6 (95.2, 97.7)	87.0 (84.7, 88.9)	78.8 (75.7, 81.7)	65.9 (61.0, 70.6)
	Xpert Ultra[†]	Sensitivity	53.8 (32.9, 74.1)	59.0 (33.9, 81.3)	59.2 (34.8, 80.7)	52.3 (25.0, 80.1)
		Specificity	99.4 (99.1, 99.5)	99.4 (99.1, 99.5)	99.4 (99.1, 99.5)	99.4 (99.1, 99.5)
	Culture	Sensitivity	75.2 (53.5, 91.3)	76.2 (49.4, 93.8)	68.1 (43.8, 89.8)	79.6 (51.4, 95.4)
		Specificity	99.8 (99.7, 99.9)	99.8 (99.7, 99.9)	99.8 (99.7, 99.9)	99.8 (99.7, 99.9)
**HIV- Female**		Prevalence	0.5 (0.3, 0.8)	0.6 (0.3, 1.0)	0.7 (0.4, 1.2)	0.6 (0.3, 1.0)
	Any TB	Sensitivity	27.8 (15.1, 43.8)	22.8 (8.6, 44.2)	27.0 (11.3, 48.7)	25.3 (8.9, 49.0)
	symptom	Specificity	81.0 (78.2, 83.6)	83.2 (80.4, 85.8)	83.1 (81.0, 85.1)	82.6 (79.7, 85.3)
	Radiologist	Sensitivity	38.5 (23.6, 55.0)	34.9 (16.8, 58.1)	48.4 (27.0, 70.4)	31.1 (13.0, 55.1)
	conclusion[Ŧ]	Specificity	99.3 (98.8, 99.7)	98.6 (97.7, 99.1)	97.9 (97.1, 98.6)	98.2 (97.2, 99.0)
	CAD4TBv5≥53	Sensitivity	74.7 (59.0, 87.2)	78.2 (56.3, 92.3)	80.2 (59.5, 93.0)	77.5 (54.3, 92.5)
		Specificity	97.8 (97.0, 98.4)	91.2 (89.2, 92.9)	84.6 (82.6, 86.5)	70.6 (67.3, 73.7)
	CAD4TBv6≥53	Sensitivity	78.7 (63.2, 89.5)	79.2 (57.8, 92.4)	83.8 (64.4, 94.6)	81.6 (59.6, 94.3)
		Specificity	98.5 (97.9, 99.0)	92.9 (91.1, 94.3)	87.2 (85.4, 88.9)	76.9 (73.8, 79.6)
	Xpert Ultra[†]	Sensitivity	57.9 (40.4, 73.9)	62.7 (38.7, 82.9)	63.2 (40.2, 82.2)	56.1 (31.4, 80.1)
		Specificity	99.4 (99.1, 99.5)	99.4 (99.1, 99.5)	99.4 (99.1, 99.5)	99.4 (99.1, 99.5)
	Culture	Sensitivity	77.6 (61.3, 89.9)	78.6 (54.8, 93.9)	71.1 (47.3, 89.6)	81.7 (59.0, 95.2)
		Specificity	99.8 (99.7, 99.9)	99.8 (99.7, 99.9)	99.8 (99.7, 99.9)	99.8 (99.7, 99.9)

Ŧ - Chest X-ray abnormality suggestive of active PTB, † - Excluding trace

Table S15: Posterior median and 95% credible intervals (95% CrI) of PTB prevalence and diagnostic test sensitivity and specificity for Vukuzazi dataset adjusted for HIV status, sex and age

		Overall[‡]	Overall[Ŧ]	Overall[†]	Overall[⊥]
	N	4960	4960	4960	4960
Test	Parameter	Median (95% CrI)	Median (95% CrI)	Median (95% CrI)	Median (95% CrI)
	Prevalence	0.9 (0.6, 1.3)	0.9 (0.6, 1.3)	1.1 (0.8, 1.5)	0.9 (0.6, 1.3)
Any TB	Sensitivity	26.7 (16.6, 38.6)	25.8 (15.9, 38.0)	24.2 (14.8, 35.8)	21.9 (12.1, 34.0)
symptom	Specificity	83.0 (81.9, 84.0)	83.0 (81.9, 84.0)	83.0 (81.9, 84.0)	82.9 (81.8, 84.0)
Radiologist	Sensitivity	84.4 (74.0, 91.9)	44.1 (32.0, 56.8)	93.2 (83.5, 98.6)	39.9 (27.1, 54.7)
conclusion	Specificity	66.7 (65.3, 68.0)	96.9 (96.3, 97.4)	66.8 (65.4, 68.1)	96.9 (96.4, 97.4)
CAD4TBv5≥53	Sensitivity	80.4 (68.4, 89.7)	81.5 (70.2, 90.2)	85.6 (72.3, 94.4)	86.7 (73.5, 95.4)
	Specificity	80.0 (78.9, 81.2)	80.1 (78.9, 81.2)	80.1 (79.0, 81.2)	80.1 (78.9, 81.2)
CAD4TBv6≥53	Sensitivity	81.1 (69.2, 90.1)	82.5 (71.3, 90.8)	84.0 (70.3, 93.3)	85.0 (72.1, 93.9)
	Specificity	82.5 (81.4, 83.6)	82.6 (81.6, 83.7)	82.6 (81.5, 83.7)	82.6 (81.5, 83.7)
Xpert Ultra	Sensitivity	62.2 (48.7, 74.4)	64.0 (50.6, 76.5)	77.2 (60.9, 89.5)	79.8 (63.8, 92.1)
	Specificity	99.4 (99.1, 99.6)	99.4 (99.1, 99.5)	99.1 (98.8, 99.4)	99.0 (98.7, 99.3)
Culture	Sensitivity	75.9 (61.9, 89.2)	75.2 (61.6, 88.0)	68.7 (54.5, 82.0)	71.7 (56.9, 85.5)
	Specificity	99.8 (99.7, 99.9)	99.8 (99.7, 99.9)	99.8 (99.7, 99.9)	99.8 (99.7, 99.9)
	Deviance	1656.8	1358.5	1692.0	1402.9

CrI – Credible Intervals

‡ - Model evaluating any TB symptom, any chest X-ray abnormality, CAD4TBv5≥53, CAD4TBv6≥53, Xpert Ultra (excluding trace) and culture

Ŧ - Model evaluating any TB symptom, chest X-ray abnormality suggestive of active PTB, CAD4TBv5≥53, CAD4TBv6≥53, Xpert Ultra (excluding trace) and culture

† - Model evaluating any TB symptom, any chest X-ray abnormality, CAD4TBv5≥53, CAD4TBv6≥53, Xpert Ultra (including trace) and culture

⊥ - Model evaluating any TB symptom, chest X-ray abnormality suggestive of active PTB, CAD4TBv5≥53, CAD4TBv6≥53, Xpert Ultra (including trace) and culture

Note: The estimates presented in the table are percentages

References

[1] Menten J, Boelaert M, Lesaffre E. Bayesian latent class models with conditionally dependent diagnostic tests: A case study. Stat Med 2008;27:4469–88. https://doi.org/10.1002/sim.3317.

[2] Albert JH, Chib S. Bayesian Analysis of Binary and Polychotomous Response Data. American Statistical Association 1993;88.

[3] Schumacher SG, van Smeden M, Dendukuri N, Joseph L, Nicol MP, Pai M, et al. Diagnostic Test Accuracy in Childhood Pulmonary Tuberculosis: A Bayesian Latent Class Analysis. Am J Epidemiol 2016;184:690–700. https://doi.org/10.1093/aje/kww094.
