# Supplementary material for: Evaluation of tuberculosis diagnostic test accuracy using Bayesian latent class analysis in the presence of conditional dependence between the diagnostic tests used in a community-based tuberculosis screening study
Source: PLoS One. 2023 Mar 2;18(3):e0282417. doi: 10.1371/journal.pone.0282417 (PMC9980779; doi:10.1371/journal.pone.0282417)
Supplement: Attachment — Submitted filename: Rebuttal Letter.pdf [file pone.0282417.s003.pdf]

Department of Clinical Sciences
TB/HIV Unit
Nationalestraat 155
2000 Antwerpen
Belgium

The Editor,
PLOS ONE

YOUR REF.	OUR REF.	ATTACHMENT(S)	ANTWERP
			20/12/2022

Subject: Edits requested on submission PONE-D-22-34380 - [EMID:ca53645f1d09b00a]

Dear The Editor,

We are pleased to provide responses to your inquiries regarding our manuscript entitled "**Evaluation of tuberculosis diagnostic test accuracy using Bayesian latent class analysis in the presence of conditional dependence between the diagnostic tests used in a community-based tuberculosis screening study**" for publication in your journal. Below are our responses.

1. Please indicate the manuscript number of the paper when it was submitted in PLOS Computational Biology. Include it in the previous interaction, at the details page.

The manuscript number at PLOS Computational Biology was PCOMPBIOL-D-22-01678 with EMID:f5e4eedd8f5191cb. This has been included in the previous interaction at the details page.

2. It has come to our attention that the manuscript seems to be a resubmission which was previously considered by the journal PLOS Computational Biology.

Thank you for this comment.

As we previously indicated during our initial submission to PLOS ONE, this manuscript was previously submitted to PLOS Computational Biology. The members of the editorial board at PLOS Computational Biology reviewed our manuscript and provided the following feedback.

"Thank you very much for submitting your manuscript "Evaluation of TB diagnostic test accuracy using Bayesian latent class analysis in the presence of conditional dependence between the diagnostic tests used in a community-based TB screening study" (PCOMPBIOL-D-22-01678) for consideration at PLOS Computational Biology. As with all papers, your manuscript was reviewed by members of the editorial board. Based on our initial assessment, we regret that we will not be pursuing this manuscript for publication at PLOS Computational Biology.

While the authors provide results related to TB, an important disease, this manuscript provides little new insight into living systems. This approach is not sufficient to warrant publication in PLOS Computational Biology. The clinical insight offered by the manuscript may better fit in a more specialized journal.

We are sorry that we cannot be more positive on this occasion. We very much appreciate your wish to present your work in one of PLOS's Open Access publications. Thank you for your support, and we hope that you will consider PLOS Computational Biology for other submissions in the future.”

We agree with the decision of the editorial board at PLOS Computational Biology that our work does not investigate the living systems. Hence, it is not suitable for publication in PLOS Computational Biology. While the editors suggested that our manuscript may be suitable for more specialized journals, we think that our work aligns well with the goals of PLOS ONE journal and will reach the wide breadth of audience and experts in the field of tuberculosis (TB) who are reading from this journal. Our manuscript considers the complications that arise in TB diagnosis due to lack of a perfect reference standard and provides an alternative approach for evaluating diagnostic tests and estimating TB prevalence.

In TB diagnosis there is no perfect reference standard. Even culture that is considered the most accurate diagnostic test suffers from an imperfect sensitivity. Hence, a negative culture test result does not rule out the presence of TB. The standard approaches using culture or a combination of culture and other microbiological tests as the reference standard to evaluate diagnostic tests and estimate TB prevalence yield biased estimates. We proposed latent class analysis (LCA) as an alternative approach. We highlight the limitations of the assumptions of LCA and suggest modifications that lead to correct estimates. We apply the proposed model to two datasets to evaluate the diagnostic test accuracy and TB prevalence. Being the first application to a community-based TB survey data, we established new findings that have not been reported elsewhere. This includes establishing the presence of dependence between any TB symptom, radiologist interpretation of chest X-ray findings and computer-Aided detection for TB test results among the true non-TB cases. We provide recommendations that will guide experts in the field of TB to ensure correct estimates are derived from TB survey data.

This manuscript addresses a fundamental problem in TB diagnosis and extends previously developed methods. It also complements some of the work already published, some in PLOS ONE. We hope that you will give a positive appraisal and allow it undergo peer review.

Changes in the manuscript from the previous version submitted to PLOS Computational Biology

There are no major changes in the manuscript from the previous version. The manuscript was formatted to align with PLOS ONE format. The only edit to the manuscript was in the title to replace the acronym “TB” by “tuberculosis”.

Thank you on behalf of the co-authors.

Alfred K. Keter

Corresponding author
